# Effects of monensin and cashew nut-shell extract on bacterial community composition in a dual-flow continuous culture system

**DOI:** 10.1093/tas/txad148

**Published:** 2023-12-23

**Authors:** Efstathios Sarmikasoglou, Phussorn Sumadong, Luiz Fernando Roesch, Sultana Halima, Chie Hikita, Tomonori Watanabe, Antonio P Faciola

**Affiliations:** Department of Animal Science, Michigan State University, East Lansing, MI 48824, USA; Department of Animal Sciences, University of Florida, Gainesville, 32611 FL, USA; Department of Animal Sciences, University of Florida, Gainesville, 32611 FL, USA; Department of Animal Science, Khon Kaen University, Khon Kaen 40002, Thailand; Department of Microbiology and Cell Science, University of Florida, Gainesville, 32603 FL, USA; Department of Animal Sciences, University of Florida, Gainesville, 32611 FL, USA; Product Development Department, SDS Biotech K.K., Tokyo 101-0022, Japan; Product Development Department, SDS Biotech K.K., Tokyo 101-0022, Japan; Department of Animal Sciences, University of Florida, Gainesville, 32611 FL, USA

**Keywords:** anacardic acid, cardol, monensin, rumen bacteria

## Abstract

The objective of this study was to evaluate the effects of including monensin and two doses of CNSE in a high producing dairy cow diet on ruminal bacterial communities. A dual-flow continuous culture system was used in a replicated 4 × 4 Latin Square design. A basal diet was formulated to meet the requirements of a cow producing 45 kg of milk per d (17% crude protein and 27% starch). There were four experimental treatments: the basal diet without any feed additive (CON), 2.5 μM monensin (MON), 100 ppm CNSE granule (CNSE100), and 200 ppm CNSE granule (CNSE200). Samples were collected from the fluid and solid effluents at 3, 6, and 9 h after feeding; a composite of all time points was made for each fermenter within their respective fractions. Bacterial community composition was analyzed by sequencing the V4 region of the 16S rRNA gene using the Illumina MiSeq platform. Treatment responses for bacterial community structure were analyzed with the PERMANOVA test run with the R *Vegan* package. Treatment responses for correlations were analyzed with the CORR procedure of SAS. Orthogonal contrasts were used to test the effects of (1) ADD (CON vs. MON, CNSE100, and CNSE200); (2) MCN (MON vs. CNSE100 and CNSE200); and (3) DOSE (CNSE100 vs. CNSE200). Significance was declared at *P *≤ 0.05. We observed that the relative abundance of *Sharpea* (*P* < 0.01)*, Mailhella* (*P* = 0.05)*, Ruminococcus* (*P* = 0.03)*, Eubacterium* (*P* = 0.01), and *Coprococcus* (*P* < 0.01) from the liquid fraction and the relative abundance of *Ruminococcus* (*P* = 0.03) and *Catonella* (*P* = 0.02) from the solid fraction decreased, while the relative abundance of *Syntrophococcus* (*P* = 0.02) increased in response to MON when compared to CNSE treatments. Our results demonstrate that CNSE and monensin have similar effects on the major ruminal bacterial genera, while some differences were observed in some minor genera. Overall, the tested additives would affect the ruminal fermentation in a similar pattern.

## Teaser

Cashew nut-shell extract affects the ruminal microbiome major bacterial genera in a similar fashion as does monensin.

## Introduction

Antibiotics, such as ionophores, and secondary plant metabolites, such as phenolic compounds, have been reported to improve animal growth and health by modulating ruminal fermentation (McGuffey et al., 2001; Calsamiglia et al., 2007; Duffield et al., 2012). Ionophores, such as monensin, and phenolic compounds, such as cashew nut-shell extract (**CNSE**), have been shown to improve ruminal fermentation by reducing amino acid deamination, increasing propionate concentration (Ruiz et al., 2001; Watanabe et al., 2010), and reducing NH_3_ and CH_4_ (Shinkai et al., 2012; Hristov et al., 2013; Knapp et al., 2014). Furthermore, monensin and CNSE have been reported to mitigate the development of metabolic disorders, such as lactic acidosis, by selectively inhibiting the growth of lactate-producing ruminal bacteria (Kubo et al., 1993; Osborne et al., 2004; Watanabe et al., 2010).

Monensin is a carboxylic polyether ionophoric antibiotic (Haney and Hoehn, 1968), which is produced by the fermentation of *Streptomyces cinnamonensis* (Anadón and Martínez-Larrañaga, 2014). Feeding monensin as a nonhormonal growth promoter in cattle (Russell and Strobel, 1989) is a common practice to improve ruminal fermentation. More specifically, monensin supplementation has been shown to inhibit H_2_-producing bacteria (Chen and Wolin, 1979), which decreases H_2_ production and availability to methanogenic archaea that require H_2_ to produce CH_4_ (McGuffey et al., 2001). Additionally, monensin has also been reported to decrease butyrate production (Russell and Strobel, 1989), as well as the rate of ruminal biohydrogenation of unsaturated fatty acids in vitro (Fellner et al., 1997), and to increase the concentration of conjugated linoleic acid in milk fat in dairy cows (AlZahal et al., 2008).

CNSE is composed of a mixture of phenolic compounds (anacardic acid, cardanol, and cardol) with selective antimicrobial (Kubo et al., 1993), and antioxidative (Kubo et al., 2006) activities. Previous studies have shown that CNSE, and particularly anacardic acid, would kill lactate-producing ruminal bacteria, such as *Streptococcus bovis*, thus reducing the accumulation of lactic acid, preventing the development of ruminal acidosis and bloat in feedlot cattle (Nagaraja and Titgemeyer, 2007; Watanabe et al., 2010; Compton, 2021), similar to the responses observed when monensin is used. Previous studies feeding 4 g of CNSE per 100 kg of BW have reported an increase in propionate production and the abundance of propionate producing bacteria (Shinkai et al., 2012), while other studies feeding CNSE at 30 g per cow per d have found no effects (Branco et al., 2015). Therefore, not only there are inconsistencies regarding CNSE dosage, but also there is a knowledge gap on the effects of CNSE on ruminal bacterial communities.

The utilization of antibiotics as feed additives has sparked concerns regarding the development of antimicrobial resistance, posing a potential threat to human health (Gadde et al., 2017); however, the effectiveness of available alternatives has been inconsistent, thus underscoring the need for further investigation of substitute solutions, in light of the worldwide imperative to decrease antibiotic usage. We hypothesized that CNSE would have similar effects to monensin on ruminal bacterial communities. Our objective was to evaluate the effects of including monensin and two doses of CNSE in a high producing dairy cow diet on ruminal bacterial communities in a dual-flow continuous culture system.

## Materials and Methods

### Ethical Approval

The University of Florida Institutional Animal Use and Care Committee approved all the animal care and handling procedures required for this experiment.

### Experimental Design, Diets, and Treatments

A detailed description of the design, diets, and treatments can be found in our companion study (Sarmikasoglou et al., 2023a). A basal diet was formulated according to the NRC, (2001) recommendations for a lactating Holstein cow with 680 kg body weight and milk production of 45 kg per d with 3.5% fat and 3.0% protein. All diets were formulated to provide the same concentration of nutrients regardless of treatment (17.1 crude protein, 4.1 ether extract, 30.3 neutral detergent fiber, and 26.8 starch, % dry matter (**DM**), (Sarmikasoglou et al., 2023a). All feed ingredients were ground through a 2-mm screen in a Wiley mill (model No. 2; Arthur H. Thomas Co., Philadelphia, PA). The corn silage was dried for 72 h at 60 °C in a forced-air oven (Heratherm, Thermo Scientific, Waltham, MA) to allow for partial dryness (>90% DM) of the material before grinding. Subsamples (500 g) from each individual ingredient were ground through a 1-mm sieve for nutrient composition analyses.

Eight fermenters of a dual-flow continuous culture system were arranged in a replicated 4 × 4 Latin Square design with a completely randomized arrangement of treatments. Treatments were (1) control without feed additives (CON); (2) monensin sodium salt (MON); (3) cashew nut-shell extract (CNSE100); and (4) double dose of cashew nut-shell extract (CNSE200).

Treatments were fed at the following concentrations: MON at 2.5 μM, final concentration in the fermenter, monensin sodium salt (M5273, Sigma-Aldrich Chemicals, Burlington, MA, USA), CNSE100 at 100 ppm and CNSE200 at 200 ppm of CNSE granule, which corresponds to 0.10 and 0.20 mg per g of DM, respectively (SDS Biotech K.K., Tokyo, Japan). The concentration of monensin was chosen based on previous dose response studies with protozoal cultures to avoid elimination of protozoa after the initial dosing so that its effect would not be limited to bacteria (Karnati et al., 2009; Sylvester et al., 2009). The concentrations of the two doses of CSNE were selected according to manufacturer guidelines and are comparable to those used previously (Watanabe et al., 2010).

Each fermenter was provided its respective experimental diet (106 g per d DM) divided equally between two feedings at 0800 and 1800 hours. The CNSE100 and CNSE200 were added as dry products to their respective diets and divided into two equal doses respectively. Regarding MON, it was used according to Sigma protocol and as established in previous studies (Sylvester et al., 2009; Capelari and Powers, 2017; Shen et al., 2017), because it is insoluble to water, MON was diluted using absolute ethanol. A stock solution (100×) was made before the experiment, stored at—20 °C, and pipetted into the fermenters immediately before both morning and evening feedings. The final ethanol concentration in the fermenters was less than 1.0 % v/v. Equal volume of absolute ethanol was pipetted to the rest fermenters to account for any effects from absolute ethanol, however potential effects of it to the physiology of the microbes cannot be excluded.

### Dual-Flow Continuous Culture System Operation

A dual-flow continuous culture system, as described by Hoover et al., (1976) and modified by Paula et al., (2017) was used for this experiment. Ruminal fermentation is simulated in this system through continuous agitation (100 rpm), infusion of N_2_ gas to displace oxygen, constant temperature (39 °C), and infusion of artificial saliva (Weller and Pilgrim, 1974) at 3.05 mL per min to individually regulate passage rates of liquid (11% per h) and solid (5.5% per h) effluents of digesta, and it has been extensively evaluated (Brandao and Faciola, 2019; Brandao et al., 2020).

This experiment consisted of 4 fermentation periods of 10 d each (40 d of in vitro fermentation total). On day 1 of each fermentation period the fermenters were inoculated with ruminal contents collected from 2 ruminally cannulated Holstein cows in mid lactation (108 ± 9 DIM on average) fed twice daily a total mixed ration with 38% corn silage, 19% ground corn, 13% soybean meal, 11% cotton seed, 9% citrus pulp, 8.5% mineral premix, and 1.5% palmitic acid supplement (on a DM basis) 3 wk before start and until completion of the experiment. Ruminal digesta was collected from the ventral, central, and dorsal areas of the rumen, strained through 2 layers of cheesecloth and transferred into prewarmed thermoses. Ruminal content from both cows was homogenized (50:50) and 1.82 L was inoculated to each fermenter. Fermenters were pre-warmed and under continuous flush of N_2_ gas during inoculation.

### Experimental Procedure and Sampling

Each experimental period consisted of four 10 d of in vitro fermentation. The first 7 d of fermentation of each period were used for adaptation to experimental diets and stabilization of bacterial communities (Salfer et al., 2018). Experimental procedure and sampling for fermentation parameters and degradability of nutrients are described in detail in Sarmikasoglou et al., (2023a).

Data and samples for bacterial sequencing from both liquid and solid effluents and main fermentation variables (ruminal pH, volatile fatty acids [**VFA**], lactate, NH_3_–N, N metabolism, and degradability measurements) were collected on days 8, 9, and 10 of each period.

Samples for bacterial sequencing analysis were collected daily from both liquid and solid effluents of each fermenter at 3, 6, and 9 h post morning feeding. For the liquid fraction, 5 mL of liquid effluent were collected at each timepoint, totaling 45 mL per fermenter per period. For the solid fraction, 22 g of solid effluent were collected at each timepoint and strained through four layers of cheesecloth, totaling ~200 g of solid sample collected from each fermenter per period. Upon collection, samples were stored at –80 °C for subsequent desoxyribonucleic acid (**DNA**) extraction.

On day 10 of each experimental period, microbial samples were collected according to the modified method of Krizsan et al. (2010) and described in detail in Brandao et al. (2018). The obtained microbial pellets were freeze-dried, grounded by mortar and pestle, and later were analyzed for ^15^N enrichment, total N and DM. All background, digesta, and microbial content samples were freeze-dried and ground with a mortar and pestle at least 24 h after the completion of freeze-drying process.

Chemical analyses for feeds, nutrients, VFA and NH_3_–N as well as N metabolism and degradability of nutrients are described in detail in Sarmikasoglou et al., (2023a).

### DNA Extraction, Polymerase Chain Reaction Amplification, and rRNA Sequencing

Total genomic DNA from ruminal samples were extracted using the Quick-DNA Fecal/Soil Microbe Miniprep Kit (D6010, Zymo Research Corporation, Irvine, CA, USA), following the manufacturer’s instructions. Before storage in −80 °C, the extracted DNA concentration was measured using a Qubit Fluorometer (Invitrogen Waltham, MA, USA). The use of a DNA extraction kit was based on the quality required for downstream analysis, consistency of the method among different lab personnel, as well as the availability of equipment, and reagents. Authors acknowledge that previously DNA extraction kits have resulted in lower community DNA recoveries from rumen content samples (Henderson et al., 2013), and potential over-/under-estimation of certain taxa are possible. However, the same method is widely used in the field with abundant data to compare to.

DNA sequencing procedures were performed according to Kozich et al. (2013). Amplification with polymerase chain reaction (**PCR**) was performed in a C1000 Touch Thermal Cycler (Bio-Rad Laboratories, Hercules, CA, USA). The V4 region of the 16S rRNA gene was amplified by dual-index universal bacterial primers (515F: 5ʹ-GTGCCAGCMGCCGCGGTAA-3ʹ; 806R: 5ʹ-GGACTACHVGGGTWTCTAAT-3ʹ; Caporaso et al., 2011) through an initial denaturation of 5 min under 95 °C, followed by 30 cycles of 30 s at 95 °C, 30 s at 55 °C, 1 min at 72 °C, and 5 min for final elongation at 72 °C. Forward and reverse primers, as well as small DNA fragment contaminants, were removed using a 1% low-melting agarose gel extraction kit. Amplicons were then purified and normalized using a SequalPrep plate kit (Invitrogen Waltham, MA, USA), and the DNA concentration was measured with a Qubit fluorometer (Invitrogen Waltham, MA, USA). Adapters were added to the amplicons, and the DNA library was constructed by equally pooling all the amplicons together and using quantitative real-time PCR for quality check. Sequencing was performed using a MiSeq reagent kit V2 (2 × 250 cycles run; Illumina) in an Illumina MiSeq platform at the Interdisciplinary Center for Biotechnology Research at the University of Florida (Gainesville, FL, USA). Sequences were deposited at the Sequence Read Archive of the National Center for Biotechnology Information (https://www.ncbi.nlm.nih.gov/sra) under access no. *PRJNA901396.*

### Bioinformatics and Analyses

Sequenced amplicons were processed using the DADA2 pipeline (version 1.16) in R (Callahan et al., 2016), and the taxonomy assignment was performed using the Bayesian RDP classifier trained with the RDP train set 18 database (Cole et al., 2014; Edgar, 2018). Briefly, paired-end raw reads were demultiplexed, and the quality profiles of the forward and reverse readings were separately inspected, filtered, and trimmed based on the relationship between error rates and quality scores. Amplicons were truncated at 30 bp to remove the forward primer. Reads with at least one ambiguous nucleotide were filtered out. The maximum error allowed in a read was set to 2. Forward and reverse readings were merged, chimeras removed, and an amplicon sequence variants table was created. The resulting tables were converted into a phyloseq object for downstream analyses. (McMurdie and Holmes, 2013). Before further data analysis, we calculated the coverage of the dataset according to Good (1953) to evaluate whether the number of sequences obtained for each sample was adequate to provide representativeness of the bacterial community (Supplementary Appendix 1). After rarefying the dataset at 10,671 sequences (equivalent to the sample with the smallest number of sequences), all samples had coverage > 99% and thus were considered representative.

To protect against amplicon sequence variants (**ASVs**) with small mean and large coefficient of variation, ASVs not seen more than 3 times in at least 20% of the samples were removed. Sequencing depth was normalized by the minimum library size (10,671 sequences per sample) to perform all microbiome analyses.

### Statistical Analyses

Bacterial alpha diversity indices (Chao1 and Shannon) were calculated with R *phyloseq* package (McMurdie and Holmes, 2013). Bacterial community structure, using the Bray–Curtis dissimilarity, was visualized by Principal Coordinates analysis (PCoA), and the statistical differences among samples were measured by PERMANOVA using the vegan R package (Oksanen, 2007). Orthogonal contrasts were used to test the effects of (1) ADD-the control compared to all treatments with additives (CON vs. MON, CNSE100 and CNSE200); (2) MCN-treatment with monensin compared to those with CNSE (MON vs. CNSE100 and CNSE200); and (3) DOSE-the single dose compared to the double dose of CNSE (CNSE100 vs. CNSE200). The orthogonal contrasts (ADD, MCN, and DOSE) were used to test the effects of the treatments on phylum, and genus differential abundance using the R *limma* package (Ritchie et al., 2015). Significance was declared at *P* ≤ 0.05, and tendencies at 0.05 < *P* ≤ 0.10. Correlations of the main fermentation variables with genera affected by the above orthogonal contrasts and relative abundance > 0.10% were analyzed using the Pearson CORR procedure of SAS. Significance was declared at *P* ≤ 0.05.

## Results and Discussion

Detailed ruminal fermentation data, including pH, total and individual VFA, N metabolism, nutrient degradability and flow, as well as protozoal numbers can be found in our companion study (Sarmikasoglou et al., 2023a).

### Effects on Rumen Bacterial Community

A total of 2,488,377 high-quality 16S rRNA sequences were retained for analysis after filtering, denoising, merging, and removing chimeras with the DADA2 pipeline. Based on those sequences, a total of 2,181 ASVs (from kingdom, phylum, class, order, family, genus) were identified after taxonomy assignment.

**Figure 1.**
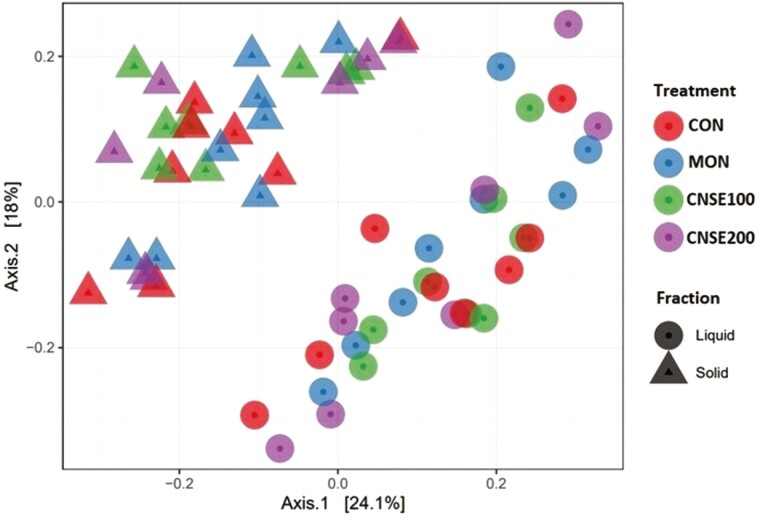
Principal coordinates analysis (PCoA) plots of Bray–Curtis dissimilarity matrix at ASV level comparing the treatment effects on community structure of ruminal bacteria. Experimental treatments were CON, control (experimental diet); MON, monensin (experimental diet plus 2.5μM monensin sodium salt); CNSE100 (experimental diet plus 100 ppm CNSE granule); CNSE200 (experimental diet plus 200 ppm CNSE granule). Contrasts were ADD = CON vs. MON, CNSE100, CNSE200; MCN = MON vs. CNSE100, CNSE200; DOSE = CNSE100 vs. CNSE200. The PERMANOVA analysis indicated that the fraction type (liquid or solid) contributed to 20% of the variation in community distances (*P* < 0.01).

The microbial community structure is reported in Figure 1. Principal coordinates analysis shows clustering in the microbiome of the liquid and solid fractions of fermentation but, no difference related to the treatments were observed regarding the community structure suggesting that the treatments did not affect the bacterial beta diversity. Moreover, the PERMANOVA analysis indicated that the fraction type (liquid or solid) contributed to 20% of the variation in community distances (*P* < 0.01). Previous studies, aligning with our findings, have highlighted the presence of bacteria in the free floating fraction co-habiting with others attached to particles (Czerkawski, 1986; Henderson et al., 2013; Monteiro et al., 2022). The analyses of treatment effects on richness and diversity at the ASV level of the bacterial community are presented in Table 1. Regarding the bacterial richness (Chao1), and diversity (Shannon), no effects were detected in any of the orthogonal contrasts tested for either liquid or solid fraction, respectively. The absence of effects suggests that the microbial diversity remains unaffected by the inclusion of monesin and/or CNSE. Previously, monensin supplementation has been reported to not affect the ruminal bacterial richness at inclusion rates of 150 and 335 mg per d in vivo (Schären et al., 2017; Melchior et al., 2018), and 5 μM (0.35 mg per mL) in vitro (Shen et al., 2017), which comes into agreement with our findings. Moreover, previous studies have reported a decrease (Schären et al., 2017; Shen et al., 2017; (Sarmikasoglou et al., 2023b) or no effect (Melchior et al., 2018) in ruminal bacterial diversity by the inclusion of monensin. Lastly, contrary to our data, previous studies found that the CNSE supplementation (4-6 g per 100 kg BW) decreased both richness and diversity (Maeda et al., 2021). Overall, it seems that monensin and CNSE function in a similar way on the structure of ruminal bacterial community; however, further research needs to elucidate their potential to affect the ruminal microbiome structure. In order to better understand the possible effects of monensin and CNSE on ruminal microbiome, we analyzed their effects on relative abundance at phylum, and genus levels.

**Table 1.** Effects of CNSE and MON in richness and diversity of the ruminal bacterial community in dual-flow continuous culture

	Treatment^1^					Contrasts *P* value^2^		
Items	CON	MON	CNSE100	CNSE200	SEM	ADD	MCN	DOSE
Liquid fraction								
Chao1	405	374	390	373	25.4	0.32	0.78	0.61
Shannon	4.57	4.41	4.52	4.51	0.10	0.26	0.17	0.94
Solid fraction								
Chao1	470	436	395	399	66.1	0.06	0.23	0.92
Shannon	5.11	5.02	4.98	4.91	0.17	0.16	0.47	0.52

^1^Experimental treatments: CON, control (experimental diet); MON, monensin (experimental diet plus 2.5μM monensin sodium salt); CNSE100 (experimental diet plus 100 ppm CNSE granule); CNSE200 (experimental diet plus 200 ppm CNSE granule).

^2^Contrasts: ADD = CON versus MON, CNSE100, CNSE200; MCN = MON versus CNSE100, CNSE200; DOSE = CNSE100 versus CNSE200.

### Effects on Bacterial Community Phyla

A total of 15 phyla were identified in both the liquid and solid fractions that exhibited a relative abundance greater than 0.10% (Table 2). The main phyla in both fractions were Firmicutes (liquid = 45.4%; solid = 53.1%), Bacteroidetes (liquid = 24.0%; solid = 26.2%), and Proteobacteria (liquid = 25.7%; solid = 13.8%), in accordance with those reported by previous studies evaluating the rumen core microbiome in cattle under predominantly forage-based diets (Fernando et al., 2010; Henderson et al., 2015; McCann et al., 2016). More specifically, greater relative abundances of Firmicutes are typically found in diets with greater forage content, while Bacteroidetes are usually predominate in diets with greater concentrate content (Clemmons et al., 2019). The major difference between fractions was the increase in the relative abundance of Proteobacteria in the liquid fraction at the expense of a small decrease in Bacteroidetes. Regarding the less-abundant phyla among those with relative abundance greater than 0.10%, Actinobacteria, Spirochaetes, Synergistetes, and Planctomycetes exhibited similar relative abundance in both fractions, while Fibrobacteres were more abundant in the solid fraction.

**Table 2.** Effects of CNSE and MON on relative abundance of ruminal bacterial community composition at phylum level

	Treatment^1^					Contrasts *P* value^2^		
Items	CON	MON	CNSE100	CNSE200	SEM	ADD	MCN	DOSE
Liquid fraction								
Firmicutes	45.9	40.3	48.7	46.6	4.01	0.84	0.12	0.23
Proteobacteria	25.7	27.6	25.1	24.7	3.15	0.95	0.50	0.49
Bacteroidetes	23.7	27.3	22.1	22.6	1.78	0.95	0.03	0.09
Actinobacteria	2.89	2.85	3.25	4.75	1.41	0.37	0.41	0.44
Spirochaetes	1.02	1.12	0.32	0.75	0.35	0.56	0.30	0.60
Synergistetes	0.23	0.32	0.24	0.21	0.05	0.78	0.28	0.29
Planctomycetes	0.22	0.17	0.2	0.2	0.07	0.49	0.76	0.58
Solid fraction								
Firmicutes	53.7	52.4	51.1	55.1	3.22	0.92	0.83	0.48
Bacteroidetes	24.7	28.9	27.5	23.8	2.02	0.27	0.10	0.03
Proteobacteria	13.8	13.1	13.6	14.8	2.12	0.92	0.76	0.71
Spirochaetes	5.00	3.20	5.47	3.63	1.83	0.40	0.68	0.91
Actinobacteria	1.28	1.46	1.15	1.80	0.32	0.54	0.72	0.75
Fibrobacteres	0.88	0.49	0.74	0.51	0.29	0.82	0.67	0.76
Planctomycetes	0.28	0.17	0.22	0.14	0.07	0.14	0.27	0.40
Synergistetes	0.14	0.18	0.18	0.10	0.03	0.85	0.22	0.04

^1^Experimental treatments: CON, control (experimental diet); MON, monensin (experimental diet plus 2.5 μ*M* monensin sodium salt); CNSE100 (experimental diet plus 100 ppm CNSE granule); CNSE200 (experimental diet plus 200 ppm CNSE granule).

^2^Contrasts: ADD = CON versus MON, CNSE100, CNSE200; MCN = MON versus CNSE100, CNSE200; DOSE = CNSE100 versus CNSE200.

In the liquid fraction, the relative abundance of Bacteroidetes was increased (MCN, *P* = 0.03), when MON compared to CNSE, while it tended to increase (DOSE, *P* = 0.09) in response to CNSE200 when compared to CNSE100. Resistance of *B. succinogenes*, and *B. ruminocola* (both belonging to Bacteroidetes phylum) to monensin has been previously observed in pure culture (Chen and Wolin, 1979), when 2.5 μg per mL of monensin was supplemented. An increase in Bacteroidetes was also reported when 368 - 518 mg per head per d of monensin was supplemented in vivo (McGarvey et al., 2019).

Concerning the solid fraction, we observed an increase in the relative abundances of Bacteroidetes (DOSE, *P* = 0.03), and Synergistetes (DOSE, *P* = 0.04), when CNSE100 compared to CNSE200. Resistance of *Ruminobacter amylophilus*, *Succinivibrio dextrinosolvens*, and *Selenomonas ruminantium* against CNSE at concentration of ≥ 50 μg per mL has been previously reported (Wakai et al., 2021), however none of those genera belong to Bacteroidetes, and/or Synergistetes. Therefore, our findings suggest that species from Bacteroidetes, and/or Synergistetes would exhibit similar effects and potential sensitivity to CNSE, when it dosed at concentrations greater than 50 μg per mL. Overall, future growth inhibition studies should focus on species from those phyla in order to assess if the development of tolerance is phylum wide or limited to species level.

### Effects on Bacterial Community Genera and Correlations Between Genera and Ruminal Fermentation Variables

To better understand changes in bacterial phyla, we conducted downstream analysis to the genus taxonomic level. Also, the genera with relative abundance > 0.10%, that had significant abundance in any of the contrasts tested, were correlated with the ruminal pH, VFA, lactate, NH_3_–N, N metabolism, and degradability measurements.

**Figure 2.**
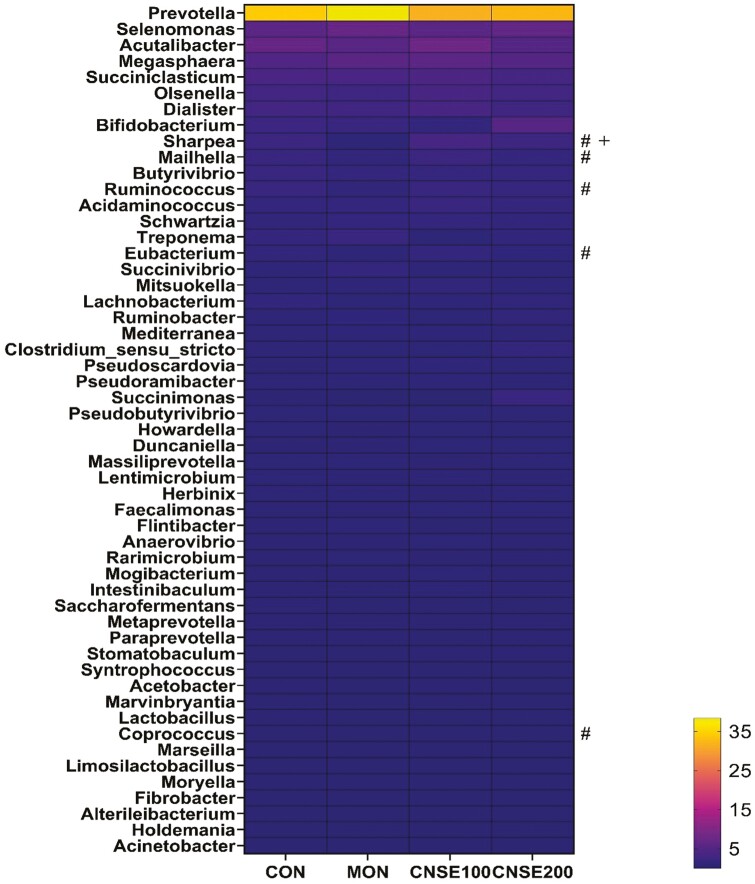
Effects of CNSE and MON on ruminal bacterial community composition in genus level in liquid fraction. Experimental treatments were CON, control (experimental diet); MON, monensin (experimental diet plus 2.5μM monensin sodium salt); CNSE100 (experimental diet plus 100 ppm CNSE granule); CNSE200 (experimental diet plus 200 ppm CNSE granule). Contrasts were ADD = CON versus MON, CNSE100, CNSE200; MCN = MON versus CNSE100, CNSE200; DOSE = CNSE100 versus CNSE200. ^#^*P*_*MCN*_ ≤ 0.05, ^+^*P*_*CNSE*_* *≤* *0.05.

**Figure 3.**
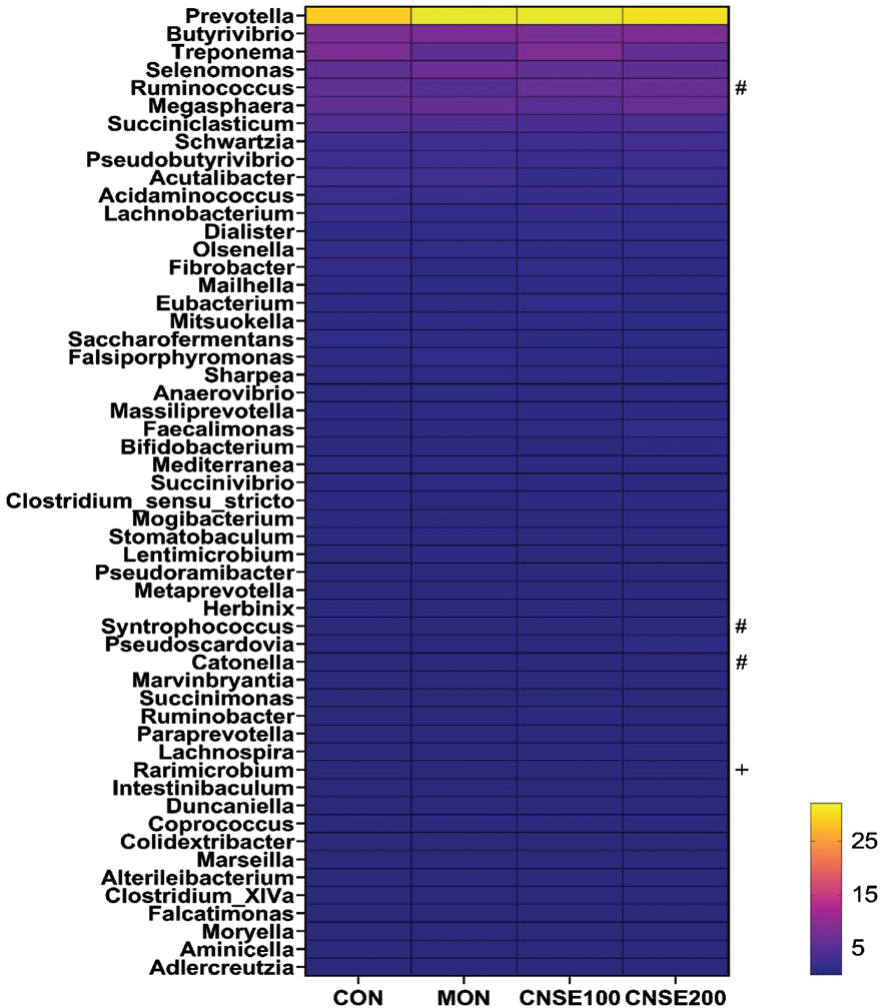
Effects of CNSE and MON on ruminal bacterial community composition in genus level in solid fraction. Experimental treatments were CON, control (experimental diet); MON, monensin (experimental diet plus 2.5μM monensin sodium salt); CNSE100 (experimental diet plus 100 ppm CNSE granule); CNSE200 (experimental diet plus 200 ppm CNSE granule). Contrasts were ADD = CON versus MON, CNSE100, CNSE200; MCN = MON versus CNSE100, CNSE200; DOSE = CNSE100 versus CNSE200. ^#^*P*_*MCN*_ ≤ 0.05, ^+^*P*_*CNSE*_* *≤* *0.05.

Based on the obtained taxa, a total of 107 out of 139 observed genera in both the liquid (Figure 2; Supplementary Appendix 2) and solid (Figure 3; Supplementary Appendix 2) fractions had a relative abundance greater than 0.10%. The total abundance (absolute) of ruminal bacteria was not affected (*P* > 0.05) by the inclusion of any of the additives tested in both fractions (data not shown). However, in order to investigate the effects of them in specific ruminal microbial populations we analyzed specific general. *Prevotella* exhibited the greatest relative abundance in both the liquid (34.0%) and the solid (30.7%) fractions. Dominance of *Prevotella* in rumen bacterial communities has been also previously reported in dual-flow continuous culture (Salfer et al., 2018; Dai et al., 2019; Monteiro et al., 2022) and in vivo (Henderson et al., 2015; Clemmons et al., 2019). *Prevotella* sensitivity to monensin has been previously observed, however some species such as *P. bryantii* seem to exhibit tolerance (Callaway and Russell, 1999, 2000; Ferme et al., 2008). Concerning the CNSE, previous reports supplemented 50 to 200 μg per mL of CNSE in vitro and reported that both in genus level (Sarmikasoglou et al., 2023b), and strain level (Watanabe et al., 2010) the relative abundance of *Prevotella* was decreased. In our study the relative abundance of *Prevotella* in liquid fraction tended to decrease (MCN, *P* = 0.07) when CNSE compared to MON (Supplementary Appendix 2), which suggests that the CNSE potential mode of action would be strain independent within *Prevotella* genus.

The relative abundance of *Sharpea* in the liquid fraction decreased (MCN, *P* < 0.01), when MON was compared to cashew treatments, while increased by CNSE100 (DOSE, *P *= 0.01), between the cashew treatments (Figure 2; Supplementary Appendix 2). The *Sharpea* spp. are anaerobic Gram-positive bacteria that belong to *Firmicutes* phylum and have been shown to produce lactate, formate, ethanol and acetate during their fermentation (Kumar et al., 2018). In general, greater abundance of *Sharpea* has been associated with low CH_4_-producing microbiomes in sheep (Kittelmann et al., 2014). Previous studies, found a decrease in *Sharpea* when haylage was supplemented with monensin at 33 mg per kg feed DM (Kim et al., 2014), which comes in accordance with our findings. Regarding CNSE, a previous study observed a decrease in *Sharpea*, when MON compared to CNSE in a serum vial culture (Sarmikasoglou et al., 2023b). Currently, no previous studies have evaluated the effects on *Sharpea* in response to CNSE; however, our results from this study and our companion study (Sarmikasoglou et al., 2023b) suggest that CNSE would promote the growth of *Sharpea*. Based on the correlations per treatment we observed a negative correlation (*r* = −0.73; *P* = 0.04) on CNSE100, between valerate concentration, and the relative abundance of *Sharpea* (Figure 5A). Regarding CNSE200, positive correlations were observed between isobutyrate (*r* = 0.74; *P* = 0.04), as well as isovalerate (*r* = 0.72; *P* = 0.04), and the relative abundance of *Sharpea*, respectively (Figure 6A). Lastly, no significant correlations were observed between MON and the fermentation variables tested. Previously, *Sharpea* has been shown a positive correlation with propionate and valerate concentrations in high milk yield (>34.5 kg per d) and high milk protein (>3.20%) content from Holstein dairy cows (Xue et al., 2019). In our study, *Sharpea* relative abundance has been shown a negative correlation with valerate under the presence of CNSE100, which would suggest that CNSE100 could potentially inhibit the *Sharpea*-enriched microbiome in a fermentation shift towards the production of intermediates, such as lactate, and end products, such as butyrate. Moreover, the relative abundance of CNSE200 was not correlated with valerate, which suggests that *Sharpea* would exhibit a non-linear correlation with it. However, further studies are needed to validate this those hypothesis. Regarding the isoforms, no previous studies have reported any correlation of isoacids with *Sharpea* in presence of cashew nut-shell. However, this study indicates that in presence of CNSE200, amino acid degradation would increase resulting in greater amounts of isobutyrate, and isovalerate. Ammonia concentration did not increase with CNSE200, which could indicate greater ammonia utilization by the microbial population.

**Figure 4.**
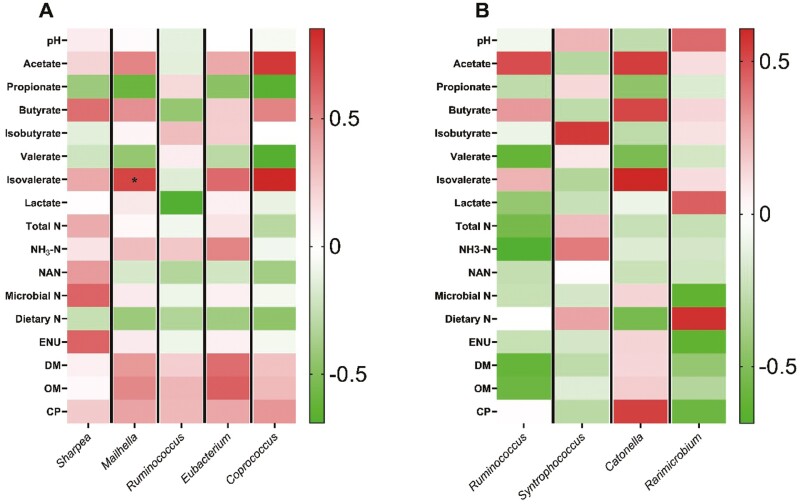
Pearson correlation analysis on MON, between the main fermentation variables and the relative abundance of the bacterial genera affected by the inclusion of the additives tested, in liquid (A), and solid (B) fractions. ^*^P ≤ 0.05.

**Figure 5.**
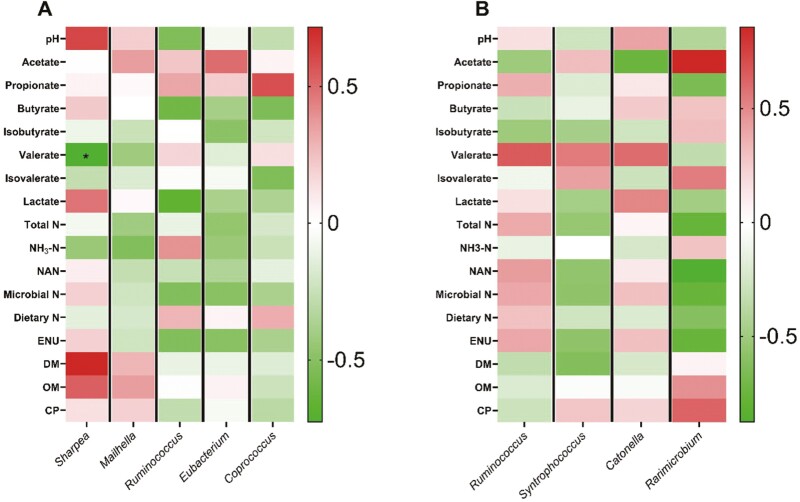
Pearson correlation analysis on CNSE100, between the main fermentation variables and the relative abundance of the bacterial genera affected by the inclusion of the additives tested, in liquid (A), and solid (B) fractions. ^*^P ≤ 0.05.

**Figure 6.**
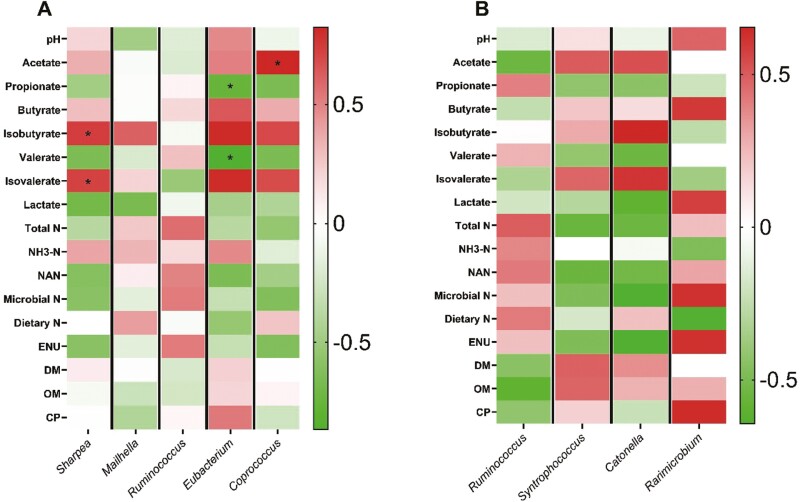
Pearson correlation analysis on CNSE200, between the main fermentation variables and the relative abundance of the bacterial genera affected by the inclusion of the additives tested, in liquid (A), and solid (B) fractions. ^*^P ≤ 0.05.

*Mailhella* relative abundance in the liquid fraction was decreased (MCN, *P *= 0.05), when MON compared to the cashew treatments (Figure 2; Supplementary Appendix 2), suggesting a potential sensitivity of this genus to ionophores. Moreover, a positive correlation (*r* = 0.73; *P* = 0.04) was observed on MON (Figure 4A), between isovalerate concentration, and the relative abundance of *Mailhella*, while no significant correlations were observed between each of the cashew treatments and the fermentation variables tested (Figures 5A and 6A).

The relative abundance of *Ruminococcus* in the liquid fraction was decreased (MCN, *P *= 0.03), when MON was compared to the cashew treatments, while tended to increase by CNSE100 (DOSE *P *= 0.10), between the cashew treatments (Figure 2; Supplementary Appendix 2). In the solid fraction the relative abundance of *Ruminococcus* decreased (MCN, *P *= 0.03) when MON compared to the cashew treatments, while tended to increase by CNSE200 (DOSE *P *= 0.06), between the cashew treatments (Figure 3; Supplementary Appendix 2). Lastly, no correlation effects were detected. Generally, *Ruminococcus* is commonly found in the rumen of ruminants and is involved in the degradation of cellulose, and starch (Xia et al., 2015; Moraïs and Mizrahi, 2019). Similar to our findings, previous studies observed a decrease on the relative abundance of *Ruminococcus* in response to monensin (Shen et al., 2017), and some *Ruminococcus* strains in response to CNSE (Watanabe et al., 2010) supplemented with dairy lactating cow diets. Overall, seems that monensin exhibits a bactericidal effect to *Ruminococcus*, while the CNSE effect seems to be strain specific.

*Eubacterium* relative abundance in the liquid fraction was decreased (MCN, *P *= 0.01), when MON compared to the cashew treatments, while tended to increase by CNSE100 (DOSE, *P *= 0.09), between the cashew treatments (Figure 2; Supplementary Appendix 2). The *Eubacterium spp.* are generally found in low levels in the rumen and primarily contribute to fiber and cellulose digestion (Kozakai et al., 2007). In addition, some *Eubacterium spp.* from high efficient cattle have been reported to produce butyrate, lactate and utilize acetate (Prins, 1971; Flint et al., 2007), as well as to be involved in the synthesis of propionate from succinate (van Gylswyk, 1995). Previous studies reported that monensin inhibits the growth of *Eubacterium ruminantium* (Dawson and Boling, 1987; Newbold et al., 2013), while others observed no effects (Kim et al., 2014; Schären et al., 2017; Sarmikasoglou et al., 2023b). Concerning the CNSE, previous pure culture studies reported that *E. ruminantium* exhibited high sensitivity to raw CNSE (Watanabe et al., 2010), while other serum vial culture studies found no effect (Sarmikasoglou et al., 2023b). Based on the correlations per treatment, we observed negative correlations on CNSE200, between propionate (*r* = −0.75; *P* = 0.03), and valerate (*r* = - 0.86; *P* = 0.01), and the relative abundance of *Eubacterium*, respectively (Figure 6A). Lastly, no significant correlations were observed between MON or CNSE100, and the fermentation variables tested (Figure 4A and B). The negative relationship of *Eubacterium* relative abundance and the concentrations of propionate and valerate on CNSE200, indicates that CNSE would inhibit the growth of *Eubacterium*, and further its respective metabolites (propionate and valerate). Those findings are in accordance with pure culture studies (Watanabe et al., 2010); however, no effects were detected in propionate or valerate in continuous culture (Sarmikasoglou et al., 2023a), potentially due to the low abundance of *Eubacterium* in the rumen.

The relative abundance of *Coprococcus* in the liquid fraction was decreased (MCN, *P *< 0.01), when MON compared to the cashew treatments, and tended to increase by CNSE100 (DOSE, *P *= 0.06), between the cashew treatments (Figure 2; Supplementary Appendix 2). Rumen isolates of *Coprococcus* has been reported to carry phloroglucinol reductase that plays a role in the degradation of phloroglucinol (Patel et al., 1981). Phloroglucinol is a phenolic compound synthesized through the degradation of flavonoids and other similar molecules, that plants normally produce and are commonly found in grazing cattle diets (Tsai et al., 1976). The function of *Coprococcus* as phloroglucinol degrader is also positively associated with the utilization of increased NADPH levels resulted from methanogenesis inhibition in the rumen. More specifically, the *Coprococcus spp.* are using NADPH as electron donor in order to reduce phloroglucinol into dihydrophloroglucinol (Patel et al., 1981), thus alleviate the elevated NADPH levels from methanogenesis inhibition (Chalupa, 1977). Except *Coprococcus* spp., the *E. oxidoreducens*, and *Streptococcus bovis* have been also identified as phloroglucinol degraders in the rumen (Patel et al., 1981; Krumholz and Bryant, 1986b). These reports would justify the similar pattern in the relative abundance between *Eubacterium* and *Coprococcus* in our study (Figure 2; Supplementary Appendix 2). Lastly, *Coprococcus* has been reported in greater abundance in the rumen of lower CH_4_ emission dairy cows by promoting the acrylate pathway for propionate production (Shabat et al., 2016), and also has been involved in the degradation of nitro-toxins in the rumen (Majak and Cheng, 1981). In our study, the CNSE is composed of a blend of phenolic compounds that seems to stimulate the growth of *Coprococcus*, thus acting similarly to flavonoids. Therefore, CNSE seems to promote the growth of species associated with the degradation of phenolic compounds, and further the utilization of NADPH, which in case of CH_4_ inhibition seems to be important. Based on the correlations per treatment, we found a positive correlation on CNSE200, between acetate (*r* = 0.82; *P* = 0.01), and the relative abundance of *Coprococcus* (Figure 6A). This finding would further indicate that CNSE would promote the growth of species associated with the degradation of phenolic compounds. Overall, further research should focus on the effects of anacardic acid, cardanol, and cardol to ruminal *Coprococcus* and assess the metabolic pathways that this genus utilizes in presence of those phenolic compounds.

*Syntrophococcus* relative abundance in the solid fraction was increased (MCN, *P *= 0.02), when MON was compared to the cashew treatments (Figure 3; Supplementary Appendix 2). Also, no correlation effects were detected (Figures 4B, 5B, and 6B). Species of *Syntrophococcus* genus are strictly anaerobic and are known to produce acetate as the end product of their fermentation (Krumholz and Bryant, 1986a). In general, acetogens are outcompeted by the methanogens in the rumen, however they are important during the initial rumen development (Morvan et al., 1994) and their association with ruminal protozoa communities (Schiel-Bengelsdorf and Dürre, 2012). Previous studies reported that *Syntrophococcus* preferentially grows in a pH = 6.00 to 7.60, increases its growth under high concentrate diets (60% to 70% grain), and has a positive correlation with caproate (Neubauer et al., 2018). Previous studies, found a decrease (Schären et al., 2017) or no effects (Kim et al., 2014) in *Syntrophococcus*, when MON supplemented in vivo. To our knowledge, no previous studies have evaluated the effects on *Syntrophococcus* in response to CNSE; however, our results suggest that CNSE would not affect growth of *Syntrophococcus*. Overall, seems that monensin exhibits a growth promoting effect to *Syntrophococcus*, while the CNSE seems to not affect it.

The relative abundance of *Catonella spp.* in the solid fraction was decreased (MCN, *P *= 0.02), when MON was compared to the cashew treatments, while tended to increase by CNSE100 (*P *= 0.09) between the cashew treatments (Figure 3; Supplementary Appendix 2). Also, no correlation effects were detected (Figures 4B, 5B, and 6B). *Catonella* belongs to the family of *Lachnospiraceae*, and has been reported that its abundance decreases drastically in presence of soybean- or fish-oil in the diet (Ngu et al., 2022). In addition, some studies have highlighted the potential positive correlation of *Catonella* with increased mean pulmonary arterial pressure, and subsequently the development of brisket disease in cattle (Gaowa et al., 2020). Currently, no previous studies have evaluated the effects of monensin, and CNSE to *Catonella* relative abundance, however, our results suggest a potential toxic effect of monensin to the genus.

The relative abundance of *Rarimicrobium* in the solid fraction decreased (DOSE, *P* = 0.04) in response to CNSE200, when compared to CNSE100, while tended to decrease (MCN, *P* = 0.10) when MON compared to the cashew treatments (Figure 3; Supplementary Appendix 2). Based on the correlations per treatment, no correlation effects were detected (Figures 4B, 5B, and 6B). The *Rarimicrobium* spp. are strictly anaerobic, non-spore forming, and nonmotile gram-negative bacteria that belongs to the phylum *Synergistetes* (Jumas-Bilak et al., 2015). Scarce knowledge about the members of *Synergistetes* exists in the literature with some studies to highlight their importance in the degradation of plant-derived toxins, and their minor participation in the fermentation of macronutrients (Allison et al., 1992). Our results indicate a potential toxic effect of phenolic compounds included in CNSE to specific species of *Synergistetes*, suggesting directions of future research.

*Lactobacillus* relative abundance (ADD, *P* = 0.07) in the liquid fraction, and the relative abundances of *Acutalibacter* (ADD, *P* = 0.09) from the solid fraction tended to decrease in response to the additives tested (Supplementary Appendix 2). Furthermore, the relative abundance of *Mitsuokella* (ADD, *P* = 0.09) from the solid fraction tended to increase in response to the additives tested (Supplementary Appendix 2). Regarding the contrast between monensin and the cashew treatments, the relative abundance of *Mediterranea* (MCN, *P* = 0.07), and *Alterileibacterium* (MCN, *P* = 0.09) in the solid fraction tended to decrease in response to cashew treatments (Supplementary Appendix 2). Concerning the comparison between the cashew treatments, the relative abundance of *Dialister* (DOSE, *P* = 0.07), and *Alterileibacterium* (DOSE, *P* = 0.05) from the solid fraction tended to increase in response to CNSE100 (Supplementary Appendix 2). Overall, except for *Lactobacillus*, the physiology and ecology of the remaining aforementioned genera is rudimentary, thus any responses from those genera do not allow for conclusive statements regarding the tested treatments.

## Conclusions

This study is one of the few that evaluated the effects of MON and CNSE on the ruminal microbiome. Most of the effects of MON and/or CNSE on the ruminal microbiome were observed on lower abundance genera, mostly in the liquid fraction of the fermentation. The relative abundance of *Sharpea, Mailhella, Ruminococcus, Eubacterium,* and *Coprococcus* in the liquid fraction, and the relative abundance of *Ruminococcus*, and *Catonella* in the solid fraction were decreased, while the relative abundance of *Syntrophococcus* was increased in response to MON when compared to CNSE treatments. Our results contribute to the current knowledge towards a better understanding of the effects of CNSE on ruminal fermentation and indicate no major differences on the ruminal microbiome pattern between MON and CNSE. Further research would be needed to compare the effects of MON, and CNSE on milk production and milk composition.

## Supplementary Material

txad148_suppl_Supplementary_Appendix

## References

[CIT0001] Allison, M. J., W. R. Mayberry, C. S. Mcsweeney, and D. A. Stahl. 1992. *Synergistes jonesii*, gen nov, sp nov: a rumen bacterium that degrades toxic pyridinediols. Syst. Appl. Microbiol. 15:522–529. doi:10.1016/s0723-2020(11)80111-6

[CIT0002] AlZahal, O., N. E. Odongo, T. Mutsvangwa, M. M. Or-Rashid, T. F. Duffield, R. Bagg, P. Dick, G. Vessie, and B. W. McBride. 2008. Effects of monensin and dietary soybean oil on milk fat percentage and milk fatty acid profile in lactating dairy cows. J. Dairy Sci. 91:1166–1174. doi:10.3168/jds.2007-0232 18292273

[CIT0003] Anadón, A., and M.R. Martínez-Larrañaga. 2014. Veterinary Drugs Residues: Coccidiostats. Y.B.T.-E. of F.S. Motarjemi, ed. Academic Press, Waltham.

[CIT0004] Branco, A. F., F. Giallongo, T. Frederick, H. Weeks, J. Oh, and A. N. Hristov. 2015. Effect of technical cashew nut shell liquid on rumen methane emission and lactation performance of dairy cows. J. Dairy Sci. 98:4030–4040. doi:10.3168/jds.2014-9015 25795493

[CIT0005] Brandao, V. L. N., and A. P. Faciola. 2019. Unveiling the relationships between diet composition and fermentation parameters response in dual-flow continuous culture system: a meta-analytical approach. Transl. Anim. Sci. 3:1064–1075. doi:10.1093/tas/txz019 32704870 PMC7200414

[CIT0006] Brandao, V. L. N., M. I. Marcondes, and A. P. Faciola. 2020. Comparison of microbial fermentation data from dual-flow continuous culture system and omasal sampling technique: a meta-analytical approach. J. Dairy Sci. 103:2347–2362. doi:10.3168/jds.2019-17107 31954580

[CIT0007] Brandao, V. L. N., L. G. Silva, E. M. Paula, H. F. Monteiro, X. Dai, A. L. J. Lelis, A. Faccenda, S. R. Poulson, and A. P. Faciola. 2018. Effects of replacing canola meal with solvent-extracted camelina meal on microbial fermentation in a dual-flow continuous culture system. J. Dairy Sci. 101:9028–9040. doi:10.3168/jds.2018-14826 30055926

[CIT0008] Callahan, B. J., P. J. McMurdie, M. J. Rosen, A. W. Han, A. J. A. Johnson, and S. P. Holmes. 2016. DADA2: High-resolution sample inference from Illumina amplicon data. Nat. Methods 13:581–583. doi:10.1038/nmeth.3869 27214047 PMC4927377

[CIT0009] Callaway, T. R., and J. B. Russell. 1999. Selection of a highly monensin-resistant Prevotella bryantii subpopulation with altered outer membrane characteristics. Appl. Environ. Microbiol. 65:4753–4759. doi:10.1128/AEM.65.11.4753-4759.1999 10543782 PMC91640

[CIT0010] Callaway, T. R., and J. B. Russell. 2000. Variations in the ability of ruminal gram-negative prevotella species to resist monensin. Curr. Microbiol. 40:185–189. doi:10.1007/s002849910037 10679051

[CIT0011] Calsamiglia, S., M. Busquet, P. W. Cardozo, L. Castillejos, and A. Ferret. 2007. Invited review: essential oils as modifiers of rumen microbial fermentation. J. Dairy Sci. 90:2580–2595. doi:10.3168/jds.2006-644 17517698

[CIT0012] Capelari, M., and W. Powers. 2017. The effect of nitrate and monensin on in vitro ruminal fermentation. J. Anim. Sci. 95:5112–5123. doi:10.2527/jas2017.1657 29293719 PMC6292307

[CIT0013] Caporaso, J. G., C. L. Lauber, W. A. Walters, D. Berg-Lyons, C. A. Lozupone, P. J. Turnbaugh, N. Fierer, and R. Knight. 2011. Global patterns of 16S rRNA diversity at a depth of millions of sequences per sample. Proc. Natl. Acad. Sci. U.S.A. 108:4516–4522. doi:10.1073/pnas.1000080107 20534432 PMC3063599

[CIT0014] Chalupa, W. 1977. Manipulating rumen fermentation. J. Anim. Sci. 45:585–599. doi:10.2527/jas1977.453585x

[CIT0015] Chen, M., and M. J. Wolin. 1979. Effect of monensin and lasalocid-sodium on the growth of methanogenic and rumen saccharolytic bacteria. Appl. Environ. Microbiol. 38:72–77. doi:10.1128/aem.38.1.72-77.1979 16345418 PMC243437

[CIT0016] Clemmons, B. A., B. H. Voy, and P. R. Myer. 2019. Altering the gut microbiome of cattle: considerations of host-microbiome interactions for persistent microbiome manipulation. Microb. Ecol. 77:523–536. doi:10.1007/s00248-018-1234-9 30033500

[CIT0017] Cole, J. R., Q. Wang, J. A. Fish, B. Chai, D. M. McGarrell, Y. Sun, C. T. Brown, A. Porras-Alfaro, C. R. Kuske, and J. M. Tiedje. 2014. Ribosomal database project: data and tools for high throughput rRNA analysis. Nucleic Acids Res. 42:D633–D642. doi:10.1093/nar/gkt1244 24288368 PMC3965039

[CIT0018] Compton, C. 2021. Effects of Cashew Nut Shell Extract on Nutrient Digestibility and Ruminal Fermentation Under in Vitro Batch Culture and Continuous Culture Conditions. All Theses. 3669 Thesis. Clemson University.

[CIT0019] Czerkawski, J.W. 1986. Degradation of solid feeds in the rumen: spatial distribution of microbial activity and its consequences. Page in Proceedings of 6th International Symposium on Ruminant Physiology, Banff (Canada), 10-14 Sep 1984. Prentice-Hall

[CIT0020] Dai, X., E. M. Paula, A. L. J. Lelis, L. G. Silva, V. L. N. Brandao, H. F. Monteiro, P. Fan, S. R. Poulson, K. C. Jeong, and A. P. Faciola. 2019. Effects of lipopolysaccharide dosing on bacterial community composition and fermentation in a dual-flow continuous culture system. J. Dairy Sci. 102:334–350. doi:10.3168/jds.2018-14807 30343924

[CIT0021] Dawson, K. A., and J. A. Boling. 1987. Effects of potassium ion concentrations on the antimicrobial activities of ionophores against ruminal anaerobes. Appl. Environ. Microbiol. 53:2363–2367. doi:10.1128/aem.53.10.2363-2367.1987 3426214 PMC204113

[CIT0022] Duffield, T. F., J. K. Merrill, and R. N. Bagg. 2012. Meta-analysis of the effects of monensin in beef cattle on feed efficiency, body weight gain, and dry matter intake. J. Anim. Sci. 90:4583–4592. doi:10.2527/jas.2011-5018 22859759

[CIT0023] Edgar, R. 2018. Taxonomy annotation and guide tree errors in 16S rRNA databases. PeerJ 6:e5030. doi:10.7717/peerj.5030 29910992 PMC6003391

[CIT0024] Fellner, V., F. D. Sauer, and J. K. Kramer. 1997. Effect of nigericin, monensin, and tetronasin on biohydrogenation in continuous flow-through ruminal fermenters. J. Dairy Sci. 80:921–928. doi:10.3168/jds.S0022-0302(97)76015-6 9178132

[CIT0025] Ferme, D., M. Malneršič, L. Lipoglavšek, C. Kamel, and G. Avguštin. 2008. Effect of sodium monensin and cinnamaldehyde on the growth and phenotypic characteristics of Prevotella bryantii and Prevotella ruminicola. Folia Microbiol. (Praha) 53:204–208. doi:10.1007/s12223-008-0026-x 18661292

[CIT0026] Fernando, S. C., H. T. Purvis, F. Z. Najar, L. O. Sukharnikov, C. R. Krehbiel, T. G. Nagaraja, B. A. Roe, and U. Desilva. 2010. Rumen microbial population dynamics during adaptation to a high-grain diet. Appl. Environ. Microbiol. 76:7482–7490. doi:10.1128/AEM.00388-10 20851965 PMC2976194

[CIT0027] Flint, H. J., S. H. Duncan, K. P. Scott, and P. Louis. 2007. Interactions and competition within the microbial community of the human colon: links between diet and health. Environ. Microbiol. 9:1101–1111. doi:10.1111/j.1462-2920.2007.01281.x 17472627

[CIT0028] Gadde, U., W. H. Kim, S. T. Oh, and H. S. Lillehoj. 2017. Alternatives to antibiotics for maximizing growth performance and feed efficiency in poultry: a review. Anim. Health Res. Rev. 18:26–45. doi:10.1017/S1466252316000207 28485263

[CIT0029] Gaowa, N., K. Panke-Buisse, S. Wang, H. Wang, Z. Cao, Y. Wang, K. Yao, and S. Li. 2020. Brisket disease is associated with lower volatile fatty acid production and altered rumen microbiome in holstein heifers. Animals. 10:1712. doi:10.3390/ani10091712 32971776 PMC7552702

[CIT0030] Good, I. J. 1953. The population frequencies of species and the estimation of population parameters. Biometrika. 40:237–264. doi:10.2307/2333344

[CIT0031] Haney, M. E., and M. M. Hoehn. 1968. Antimicrobial agents and chemotherapy–1967. Ann Arbor 7:349–352. doi:10.1128/AAC.7.3.349. 5596158

[CIT0032] Henderson, G., F. Cox, S. Ganesh, A. Jonker, W. Young, L. Abecia, E. Angarita, P. Aravena, G. Nora Arenas, C. Ariza, et al. 2015. Rumen microbial community composition varies with diet and host, but a core microbiome is found across a wide geographical range. Sci. Rep. 5: 14567. doi:10.1038/srep14567 26449758 PMC4598811

[CIT0033] Henderson, G., F. Cox, S. Kittelmann, V. H. Miri, M. Zethof, S. J. Noel, G. C. Waghorn, and P. H. Janssen. 2013. Effect of DNA extraction methods and sampling techniques on the apparent structure of cow and sheep rumen microbial communities. PLoS One 8:e74787. doi:10.1371/journal.pone.0074787 24040342 PMC3770609

[CIT0034] Hristov, A. N., J. Oh, J. L. Firkins, J. Dijkstra, E. Kebreab, G. Waghorn, H. P. S. Makkar, A. T. Adesogan, W. Yang, C. Lee, et al. 2013. Special Topics—mitigation of methane and nitrous oxide emissions from animal operations: I. A review of enteric methane mitigation options. J. Anim. Sci. 91:5045–5069. doi:10.2527/jas.2013-6583 24045497

[CIT0085] Hoover, W. H., Crooker, B. A., and Sniffen. C. J. 1976. Effects of differential solid-liquid removal rates on protozoa numbers in continous cultures of rumen contents. J. Anim. Sci. 43:528–534. doi:10.2527/jas1976.432528x.

[CIT0035] Jumas-Bilak, E., P. Bouvet, E. Allen-Vercoe, F. Aujoulat, P. A. Lawson, H. Jean-Pierre, and H. Marchandin. 2015. Rarimicrobium hominis gen nov, sp nov, representing the fifth genus in the phylum Synergistetes that includes human clinical isolates. Int. J. Syst. Evol. Microbiol. 65:3965–3970. doi:10.1099/ijsem.0.000520 26320053

[CIT0036] Karnati, S. K. R., J. T. Sylvester, C. V. D. M. Ribeiro, L. E. Gilligan, and J. L. Firkins. 2009. Investigating unsaturated fat, monensin, or bromoethanesulfonate in continuous cultures retaining ruminal protozoa. I. Fermentation, biohydrogenation, and microbial protein synthesis. J. Dairy Sci. 92:3849–3860. doi:10.3168/jds.2008-1436 19620669

[CIT0037] Kim, M., T. L. Felix, S. C. Loerch, and Z. Yu. 2014. Effect of haylage and monensin supplementation on ruminal bacterial communities of feedlot cattle. Curr. Microbiol. 69:169–175. doi:10.1007/s00284-014-0564-1 24682259

[CIT0038] Kittelmann, S., C. S. Pinares-Patiño, H. Seedorf, M. R. Kirk, S. Ganesh, J. C. McEwan, and P. H. Janssen. 2014. Two different bacterial community types are linked with the low-methane emission trait in sheep. PLoS One 9:e103171. doi:10.1371/journal.pone.0103171 25078564 PMC4117531

[CIT0039] Knapp, J. R., G. L. Laur, P. A. Vadas, W. P. Weiss, and J. M. Tricarico. 2014. Invited review: enteric methane in dairy cattle production: quantifying the opportunities and impact of reducing emissions. J. Dairy Sci. 97:3231–3261. doi:10.3168/jds.2013-7234 24746124

[CIT0040] Kozakai, K., T. Nakamura, Y. Kobayashi, T. Tanigawa, I. Osaka, S. Kawamoto, and S. Hara. 2007. Effect of mechanical processing of corn silage on in vitro ruminal fermentation, and in situ bacterial colonization and dry matter degradation. Can. J. Anim. Sci. 87:259–267. doi:10.4141/a06-028

[CIT0041] Kozich, J. J., S. L. Westcott, N. T. Baxter, S. K. Highlander, and P. D. Schloss. 2013. Development of a dual-index sequencing strategy and curation pipeline for analyzing amplicon sequence data on the miseq illumina sequencing platform. Appl. Environ. Microbiol. 79:5112–5120. doi:10.1128/aem.01043-13 23793624 PMC3753973

[CIT0042] Krizsan, S. J., S. Ahvenjärvi, H. Volden, and G. A. Broderick. 2010. Estimation of rumen outflow in dairy cows fed grass silage-based diets by use of reticular sampling as an alternative to sampling from the omasal canal. J. Dairy Sci. 93:1138–1147. doi:10.3168/jds.2009-2661 20172235

[CIT0043] Krumholz, L., and M. Bryant. 1986a. *Syntrophococcus sucromutans* sp. nov. gen nov. uses carbohydrates as electron donors and formate, methoxymonobenzenoids or *Methanobrevibacter* as electron acceptor systems. Arch. Microbiol. 143:313–318. doi:10.1007/BF00412795.

[CIT0044] Krumholz, L. R., and M. Bryant. 1986b. *Eubacterium oxidoreducens* sp. nov. requiring H 2 or formate to degrade gallate, pyrogallol, phloroglucinol and quercetin. Arch. Microbiol. 144:8–14. doi:10.1007/BF00454948.

[CIT0045] Kubo, I., M. Hisae, H. Masaki, Y. Yoshiro, M. Hiroyuki, T. Kimihiro, O. Shigeo, and K. Tadao. 1993. Structure-antibacterial activity relationships of anacardic acids. J. Agric. Food Chem. 41:1016–1019. doi:10.1021/jf00030a036

[CIT0046] Kubo, I., N. Masuoka, T. J. Ha, and K. Tsujimoto. 2006. Antioxidant activity of anacardic acids. Food Chem. 99:555–562. doi:10.1016/j.foodchem.2005.08.023

[CIT0047] Kumar, S., B. P. Treloar, K. H. Teh, C. M. McKenzie, G. Henderson, G. T. Attwood, S. M. Waters, M. L. Patchett, and P. H. Janssen. 2018. Sharpea and Kandleria are lactic acid producing rumen bacteria that do not change their fermentation products when co-cultured with a methanogen. Anaerobe 54:31–38. doi:10.1016/j.anaerobe.2018.07.008 30055268

[CIT0048] Maeda, K., V. T. Nguyen, T. Suzuki, K. Yamada, K. Kudo, C. Hikita, V. P. Le, M. C. Nguyen, and N. Yoshida. 2021. Network analysis and functional estimation of the microbiome reveal the effects of cashew nut shell liquid feeding on methanogen behaviour in the rumen. Microb. Biotechnol. 14:277–290. doi:10.1111/1751-7915.13702 33166077 PMC7888476

[CIT0049] Majak, W., and K. J. Cheng. 1981. Identification of rumen bacteria that anaerobically degrade aliphatic nitrotoxins. Can. J. Microbiol. 27:646–650. doi:10.1139/m81-099 7197575

[CIT0050] McCann, J. C., S. Luan, F. C. Cardoso, H. Derakhshani, E. Khafipour, and J. J. Loor. 2016. Induction of subacute ruminal acidosis affects the ruminal microbiome and epithelium. Front. Microbiol. 7:701–701. doi:10.3389/fmicb.2016.00701 27242724 PMC4870271

[CIT0051] McGarvey, J. A., S. Place, J. Palumbo, R. Hnasko, and F. Mitloehner. 2019. Dosage-dependent effects of monensin on the rumen microbiota of lactating dairy cattle. MicrobiologyOpen 8:e00783. doi:10.1002/mbo3.783 30565435 PMC6612561

[CIT0052] McGuffey, R. K., L. F. Richardson, and J. I. D. Wilkinson. 2001. Ionophores for dairy cattle: current status and future outlook. J. Dairy Sci. 84:E194–E203. doi:10.3168/jds.s0022-0302(01)70218-4

[CIT0053] McMurdie, P. J., and S. Holmes. 2013. phyloseq: An R package for reproducible interactive analysis and graphics of microbiome census data. PLoS One 8:e61217. doi:10.1371/journal.pone.0061217 23630581 PMC3632530

[CIT0054] Melchior, E. A., K. E. Hales, A. K. Lindholm-Perry, H. C. Freetly, J. E. Wells, C. N. Hemphill, T. A. Wickersham, J. E. Sawyer, and P. R. Myer. 2018. The effects of feeding monensin on rumen microbial communities and methanogenesis in bred heifers fed in a drylot. Livest. Sci. 212:131–136. doi:10.1016/j.livsci.2018.03.019

[CIT0055] Monteiro, H. F., A. L. J. Lelis, P. Fan, B. Calvo Agustinho, R. R. Lobo, J. A. Arce-Cordero, X. Dai, K. C. Jeong, and A. P. Faciola. 2022. Effects of lactic acid-producing bacteria as direct-fed microbials on the ruminal microbiome. J. Dairy Sci. 105:2242–2255. doi:10.3168/jds.2021-21025 34998552

[CIT0056] Moraïs, S., and I. Mizrahi. 2019. The road not taken: the rumen microbiome, functional groups, and community states. Trends Microbiol. 27:538–549. doi:10.1016/j.tim.2018.12.011 30679075

[CIT0057] Morvan, B., J. Dore, F. Rieu-Lesme, L. Foucat, G. Fonty, and P. Gouet. 1994. Establishment of hydrogen-utilizing bacteria in the rumen of the newborn lamb. FEMS Microbiol. Lett. 117:249–256. doi:10.1016/0378-1097(94)90567-3 8200502

[CIT0058] Nagaraja, T. G., and E. C. Titgemeyer. 2007. Ruminal acidosis in beef cattle: the current microbiological and nutritional outlook. J. Dairy Sci. 90:E17–E38. doi:10.3168/jds.2006-478 17517750

[CIT0059] Neubauer, V., R. Petri, E. Humer, I. Kröger, E. Mann, N. Reisinger, M. Wagner, and Q. Zebeli. 2018. High-grain diets supplemented with phytogenic compounds or autolyzed yeast modulate ruminal bacterial community and fermentation in dry cows. J. Dairy Sci. 101:2335–2349. doi:10.3168/jds.2017-13565 29331466

[CIT0060] Newbold, C. J., R. J. Wallace, and N. D. Walker-Bax. 2013. Potentiation by metal ions of the efficacy of the ionophores, monensin and tetronasin, towards four species of ruminal bacteria. FEMS Microbiol. Lett. 338:161–167. doi:10.1111/1574-6968.12044 23210858

[CIT0061] Ngu, N. T., L. H. Anh, N. T. H. Nhan, N. Van Hon, N. Thiet, J. B. Liang, L. T. Hung, N. H. Xuan, W. L. Chen, and L. T. T. Lan. 2022. Analysis of bacterial community in rumen fluid of cattle supplemented with different protein and energy sources. Anim. Prod. Sci. 62:1353–1361. doi:10.1071/an20206

[CIT0087] NRC. 2001. Nutrient Requirements of Dairy Cattle: 2001. National Academies Press. 38386771

[CIT0062] Oksanen, J. 2007. Vegan: community ecology package. R package version 1.8-5. https://cran.r-project.org/

[CIT0063] Osborne, J. K., T. Mutsvangwa, O. Alzahal, T. F. Duffield, R. Bagg, P. Dick, G. Vessie, and B. W. McBride. 2004. Effects of monensin on ruminal forage degradability and total tract diet digestibility in lactating dairy cows during grain-induced subacute ruminal acidosis. J. Dairy Sci. 87:1840–1847. doi:10.3168/jds.S0022-0302(04)73341-X 15453500

[CIT0064] Patel, T. R., K. G. Jure, and G. A. Jones. 1981. Catabolism of phloroglucinol by the rumen anaerobe coprococcus. Appl. Environ. Microbiol. 42:1010–1017. doi:10.1128/aem.42.6.1010-1017.1981 16345897 PMC244147

[CIT0086] Paula, E.M., Monteiro, H.F., Silva, L.G., Benedeti, P.D.B., Daniel, J.L.P., Shenkoru, T., Broderick, G.A., Faciola. A.P. 2017. Effects of replacing soybean meal with canola meal differing in rumen-undegradable protein content on ruminal fermentation and gas production kinetics using 2 in vitro systems. J. Dairy Sci. 100:5281-5292. 28456405 10.3168/jds.2016-12301

[CIT0065] Prins, R. A. 1971. Isolation, culture, and fermentation characteristics of Selenomonas ruminantium var. bryantivar. n. from the rumen of sheep. J. Bacteriol. 105:820–825. doi:10.1128/jb.105.3.820-825.1971 4323298 PMC248505

[CIT0066] Ritchie, M. E., B. Phipson, D. Wu, Y. Hu, C. W. Law, W. Shi, and G. K. Smyth. 2015. Limma powers differential expression analyses for RNA-sequencing and microarray studies. Nucleic Acids Res. 43:e47. doi:10.1093/nar/gkv007 25605792 PMC4402510

[CIT0067] Ruiz, R., G. L. Albrecht, L. O. Tedeschi, G. Jarvis, J. B. Russell, and D. G. Fox. 2001. Effect of monensin on the performance and nitrogen utilization of lactating dairy cows consuming fresh forage. J. Dairy Sci. 84:1717–1727. doi:10.3168/jds.S0022-0302(01)74607-3 11467822

[CIT0068] Russell, J. B., and H. J. Strobel. 1989. Effect of ionophores on ruminal fermentation. Appl. Environ. Microbiol. 55:1–6. doi:10.1128/aem.55.1.1-6.1989 2650616 PMC184044

[CIT0083] Sarmikasoglou, E., Johnson, M.L., Vinyard, J.R., Sumadong, P., Lobo, R.R., Arce-Cordero, J.A., Bahman, A., Ravelo, A., Halima, S., Salas-Solis, G.K., et al. 2023a. Effects of cashew nutshell extract and monensin on microbial fermentation in a dual-flow continuous culture. J. Dairy Sci. 106 (12):8746–8757. 37678783 10.3168/jds.2023-23597

[CIT0084] Sarmikasoglou, E., Sumadong, P., Roesch, L.F.W., Halima, S., Arriola, K., Yuting, Z., Jeong, K.C.C., Vyas, D., Hikita, C., Watanabe, T., et al. 2023.Effects of cashew nut-shell extract and monensin on in vitro ruminal fermentation, methane production, and ruminal bacterial community. J. Dairy Sci. 10.3168/jds.2023-23669 37730175

[CIT0069] Salfer, I. J., C. Staley, H. E. Johnson, M. J. Sadowsky, and M. D. Stern. 2018. Comparisons of bacterial and archaeal communities in the rumen and a dual-flow continuous culture fermentation system using amplicon sequencing. J. Anim. Sci. 96:1059–1072. doi:10.1093/jas/skx056 29529208 PMC6093571

[CIT0070] Schären, M., C. Drong, K. Kiri, S. Riede, M. Gardener, U. Meyer, J. Hummel, T. Urich, G. Breves, and S. Dänicke. 2017. Differential effects of monensin and a blend of essential oils on rumen microbiota composition of transition dairy cows. J. Dairy Sci. 100:2765–2783. doi:10.3168/jds.2016-11994 28161182

[CIT0071] Schiel-Bengelsdorf, B., and P. Dürre. 2012. Pathway engineering and synthetic biology using acetogens. FEBS Lett. 586:2191–2198. doi:10.1016/j.febslet.2012.04.043 22710156

[CIT0072] Shabat, S. K. B., G. Sasson, A. Doron-Faigenboim, T. Durman, S. Yaacoby, M. E. Berg Miller, B. A. White, N. Shterzer, and I. Mizrahi. 2016. Specific microbiome-dependent mechanisms underlie the energy harvest efficiency of ruminants. ISME J. 10:2958–2972. doi:10.1038/ismej.2016.62 27152936 PMC5148187

[CIT0073] Shen, J., Z. Liu, Z. Yu, and W. Zhu. 2017. Monensin and nisin affect rumen fermentation and microbiota differently in vitro. Front. Microbiol. 8:1111. doi:10.3389/fmicb.2017.01111 28670304 PMC5472720

[CIT0074] Shinkai, T., O. Enishi, M. Mitsumori, K. Higuchi, Y. Kobayashi, A. Takenaka, K. Nagashima, M. Mochizuki, and Y. Kobayashi. 2012. Mitigation of methane production from cattle by feeding cashew nut shell liquid. J. Dairy Sci. 95:5308–5316. doi:10.3168/jds.2012-5554 22916936

[CIT0075] Sylvester, J. T., S. K. R. Karnati, B. A. Dehority, M. Morrison, G. L. Smith, N. R. St-Pierre, and J. L. Firkins. 2009. Rumen ciliated protozoa decrease generation time and adjust 18S ribosomal DNA copies to adapt to decreased transfer interval, starvation, and monensin. J. Dairy Sci. 92:256–269. doi:10.3168/jds.2008-1417 19109285

[CIT0076] Tsai, C. G., D. M. Gates, W. M. Ingledew, and G. A. Jones. 1976. Products of anaerobic phloroglucinol degradation by Coprococcus sp. Pe15. Can. J. Microbiol. 22:159–164. doi:10.1139/m76-022 944077

[CIT0077] van Gylswyk, N. O. 1995. Succiniclasticum ruminis gen. nov., sp. nov., a ruminal bacterium converting succinate to propionate as the sole energy-yielding mechanism. Int. J. Syst. Bacteriol. 45:297–300. doi:10.1099/00207713-45-2-297 7537062

[CIT0078] Wakai, M., S. Hayashi, Y. Chiba, S. Koike, K. Nagashima, and Y. Kobayashi. 2021. Growth and morphologic response of rumen methanogenic archaea and bacteria to cashew nut shell liquid and its alkylphenol components. Anim. Sci. J. 92:e13598. doi:10.1111/asj.13598 34350672

[CIT0079] Watanabe, Y., R. Suzuki, S. Koike, K. Nagashima, M. Mochizuki, R. J. Forster, and Y. Kobayashi. 2010. In vitro evaluation of cashew nut shell liquid as a methane-inhibiting and propionate-enhancing agent for ruminants. J. Dairy Sci. 93:5258–5267. doi:10.3168/jds.2009-2754 20965342

[CIT0080] Weller, R. A., A. F. Pilgrim. 1974. Passage of protozoa and volatile fatty acids from the rumen of the sheep and from a continuous in vitro fermentation system. Br. J. Nutr. 32:341–351. doi:10.1079/BJN19740087 4213614

[CIT0081] Xia, Y., Y. Kong, R. Seviour, H. -E. Yang, R. Forster, T. Vasanthan, and T. McAllister. 2015. In situ identification and quantification of starch-hydrolyzing bacteria attached to barley and corn grain in the rumen of cows fed barley-based diets. FEMS Microbiol. Ecol. 91:fiv077. doi:10.1093/femsec/fiv077 26142428

[CIT0082] Xue, M. Y., H. Z. Sun, X. H. Wu, L. L. Guan, and J. X. Liu. 2019. Assessment of rumen bacteria in dairy cows with varied milk protein yield. J. Dairy Sci. 102:5031–5041. doi:10.3168/jds.2018-15974 30981485

