# Supplementary material for: Effects of monensin and cashew nut-shell extract on bacterial community composition in a dual-flow continuous culture system
Source: Transl Anim Sci. 2023 Dec 23;8:txad148. doi: 10.1093/tas/txad148 (PMC10787353; doi:10.1093/tas/txad148)
Supplement: txad148_suppl_Supplementary_Appendix [file txad148_suppl_supplementary_appendix.docx]

APPENDIX 1: Distribution of reads per sample.

| Sample | Treatment | Total Reads | Rarefied Reads | Coverage after rarefaction |
| --- | --- | --- | --- | --- |
| ES37-SA701-SA504 | CON | 26707 | 10671 | 0.998501 |
| ES41-SA705-SA504 | CON | 64499 | 10671 | 0.995221 |
| ES48-SA712-SA504 | CON | 19389 | 10671 | 0.998782 |
| ES50-SA702-SA505 | CON | 39797 | 10671 | 0.996252 |
| ES55-SA707-SA505 | CON | 41331 | 10671 | 0.99672 |
| ES59-SA711-SA505 | CON | 42925 | 10671 | 0.996814 |
| ES62-SA702-SA506 | CON | 63073 | 10671 | 0.99344 |
| ES68-SA708-SA506 | CON | 40732 | 10671 | 0.996345 |
| ES69-SA709-SA506 | CON | 25740 | 10671 | 0.998688 |
| ES73-SA701-SA507 | CON | 24251 | 10671 | 0.998782 |
| ES80-SA708-SA507 | CON | 27617 | 10671 | 0.998313 |
| ES82-SA710-SA507 | CON | 30986 | 10671 | 0.997751 |
| ES87-SA703-SA508 | CON | 32949 | 10671 | 0.997189 |
| ES91-SA707-SA508 | CON | 67738 | 10671 | 0.99419 |
| ES94-SA710-SA508 | CON | 51044 | 10671 | 0.995502 |
| S62-SA711-SB506 | CON | 69031 | 10671 | 0.994471 |
| ES40-SA704-SA504 | MON | 53367 | 10671 | 0.994096 |
| ES44-SA708-SA504 | MON | 85891 | 10671 | 0.995596 |
| ES47-SA711-SA504 | MON | 23670 | 10671 | 0.998688 |
| ES49-SA701-SA505 | MON | 20416 | 10671 | 0.998688 |
| ES54-SA706-SA505 | MON | 45772 | 10671 | 0.997564 |
| ES58-SA710-SA505 | MON | 33521 | 10671 | 0.998032 |
| ES61-SA701-SA506 | MON | 28357 | 10671 | 0.998688 |
| ES67-SA707-SA506 | MON | 47482 | 10671 | 0.996533 |
| ES72-SA712-SA506 | MON | 37047 | 10671 | 0.997189 |
| ES76-SA704-SA507 | MON | 25539 | 10671 | 0.99925 |
| ES79-SA707-SA507 | MON | 24007 | 10671 | 0.998782 |
| ES81-SA709-SA507 | MON | 23281 | 10671 | 0.998969 |
| ES86-SA702-SA508 | MON | 83433 | 10671 | 0.992784 |
| ES90-SA706-SA508 | MON | 43965 | 10671 | 0.997095 |
| ES93-SA709-SA508 | MON | 59368 | 10671 | 0.995314 |
| S61-SA712-SB506 | MON | 36238 | 10671 | 0.996908 |
| ES38-SA702-SA504 | CNSE100 | 34370 | 10671 | 0.997564 |
| ES42-SA706-SA504 | CNSE100 | 46425 | 10671 | 0.996814 |
| ES45-SA709-SA504 | CNSE100 | 50827 | 10671 | 0.997001 |
| ES51-SA703-SA505 | CNSE100 | 48207 | 10671 | 0.996814 |
| ES56-SA708-SA505 | CNSE100 | 59826 | 10671 | 0.995127 |
| ES60-SA712-SA505 | CNSE100 | 21918 | 10671 | 0.999063 |
| ES63-SA703-SA506 | CNSE100 | 53566 | 10671 | 0.996064 |
| ES65-SA705-SA506 | CNSE100 | 42659 | 10671 | 0.996064 |
| ES70-SA710-SA506 | CNSE100 | 31802 | 10671 | 0.99822 |
| ES74-SA702-SA507 | CNSE100 | 30314 | 10671 | 0.998876 |
| ES77-SA705-SA507 | CNSE100 | 28067 | 10671 | 0.999063 |
| ES83-SA711-SA507 | CNSE100 | 15579 | 10671 | 0.999719 |
| ES88-SA704-SA508 | CNSE100 | 53117 | 10671 | 0.995502 |
| ES92-SA708-SA508 | CNSE100 | 48431 | 10671 | 0.995877 |
| ES95-SA711-SA508 | CNSE100 | 36094 | 10671 | 0.998501 |
| S59-SA702-SB505 | CNSE100 | 26714 | 10671 | 0.998501 |
| ES39-SA703-SA504 | CNSE200 | 47236 | 10671 | 0.997564 |
| ES43-SA707-SA504 | CNSE200 | 43241 | 10671 | 0.998032 |
| ES46-SA710-SA504 | CNSE200 | 27420 | 10671 | 0.998594 |
| ES52-SA704-SA505 | CNSE200 | 47815 | 10671 | 0.995783 |
| ES53-SA705-SA505 | CNSE200 | 40543 | 10671 | 0.997001 |
| ES57-SA709-SA505 | CNSE200 | 36828 | 10671 | 0.996626 |
| ES64-SA704-SA506 | CNSE200 | 30678 | 10671 | 0.997095 |
| ES66-SA706-SA506 | CNSE200 | 23440 | 10671 | 0.998126 |
| ES71-SA711-SA506 | CNSE200 | 20334 | 10671 | 0.998876 |
| ES75-SA703-SA507 | CNSE200 | 32789 | 10671 | 0.998407 |
| ES78-SA706-SA507 | CNSE200 | 27500 | 10671 | 0.998594 |
| ES85-SA701-SA508 | CNSE200 | 43011 | 10671 | 0.996158 |
| ES89-SA705-SA508 | CNSE200 | 58844 | 10671 | 0.994752 |
| S58-SA703-SB505 | CNSE200 | 30948 | 10671 | 0.997751 |
| S60-SA701-SB505 | CNSE200 | 10671 | 10671 | 1 |

APPENDIX 2: Effects of CNSE and MON on relative abundance of ruminal bacterial community composition in genus level

|  | Treatment^1^ | | | |  | Contrasts *P*-value^2^ | | |
| --- | --- | --- | --- | --- | --- | --- | --- | --- |
| Items | CON | MON | CNSE100 | CNSE200 | SEM | ADD | MCN | DOSE |
| Liquid Fraction |  |  |  |  |  |  |  |  |
| *Prevotella* | 34.1 | 38.4 | 31.4 | 32.1 | 3.34 | 0.92 | 0.07 | 0.13 |
| *Selenomonas* | 6.07 | 7.43 | 5.85 | 6.70 | 0.99 | 0.74 | 0.33 | 0.54 |
| *Acutalibacter* | 7.08 | 5.60 | 8.05 | 5.19 | 1.32 | 0.20 | 0.46 | 0.97 |
| *Megasphaera* | 4.64 | 5.74 | 5.88 | 5.23 | 1.03 | 0.54 | 0.94 | 0.99 |
| *Succiniclasticum* | 3.84 | 3.86 | 4.19 | 3.35 | 0.68 | 0.97 | 0.80 | 0.46 |
| *Olsenella* | 3.08 | 2.63 | 3.71 | 2.93 | 0.99 | 0.71 | 0.34 | 0.57 |
| *Dialister* | 3.02 | 2.75 | 3.7 | 2.70 | 0.75 | 0.83 | 0.83 | 0.35 |
| *Bifidobacterium* | 2.26 | 2.13 | 1.56 | 5.37 | 1.59 | 0.79 | 0.74 | 0.57 |
| *Sharpea* | 2.32 | 0.94 | 3.48 | 2.47 | 0.35 | 0.55 | < 0.01 | 0.01 |
| *Mailhella* | 2.08 | 1.46 | 2.45 | 1.79 | 0.36 | 0.43 | 0.05 | 0.15 |
| *Butyrivibrio* | 2.22 | 1.74 | 1.87 | 1.93 | 0.34 | 0.26 | 0.77 | 0.65 |
| *Ruminococcus* | 2.04 | 1.46 | 2.06 | 1.92 | 0.24 | 0.28 | 0.03 | 0.10 |
| *Acidaminococcus* | 1.61 | 1.64 | 2.05 | 1.77 | 0.28 | 0.56 | 0.53 | 0.91 |
| *Schwartzia* | 1.47 | 1.60 | 1.65 | 1.42 | 0.20 | 0.78 | 0.61 | 0.58 |
| *Treponema* | 1.79 | 2.05 | 0.60 | 1.42 | 0.60 | 0.48 | 0.25 | 0.54 |
| *Eubacterium* | 1.36 | 0.92 | 1.68 | 1.23 | 0.24 | 0.93 | 0.01 | 0.09 |
| *Succinivibrio* | 1.18 | 1.62 | 0.98 | 1.08 | 0.45 | 0.60 | 0.13 | 0.21 |
| *Mitsuokella* | 0.99 | 1.21 | 1.29 | 1.21 | 0.23 | 0.28 | 0.86 | 0.96 |
| *Lachnobacterium* | 1.37 | 0.83 | 1.13 | 1.32 | 0.21 | 0.21 | 0.30 | 0.24 |
| *Ruminobacter* | 0.94 | 1.23 | 0.67 | 1.35 | 0.63 | 0.97 | 0.25 | 0.69 |
| *Mediterranea* | 0.98 | 1.09 | 1.08 | 0.99 | 0.14 | 0.97 | 0.78 | 0.51 |
| *Clostridium_sensu_stricto* | 0.98 | 0.89 | 0.68 | 1.52 | 0.86 | 0.69 | 0.75 | 0.97 |
| *Pseudoscardovia* | 0.52 | 1.05 | 1.18 | 0.72 | 0.38 | 0.69 | 0.89 | 0.70 |
| *Pseudoramibacter* | 0.88 | 0.73 | 1.00 | 0.80 | 0.31 | 0.63 | 0.56 | 0.99 |
| *Succinimonas* | 0.47 | 0.28 | 0.01 | 2.11 | 0.96 | 0.15 | 0.88 | 0.46 |
| *Pseudobutyrivibrio* | 0.82 | 0.57 | 0.57 | 0.72 | 0.16 | 0.17 | 0.84 | 0.63 |
| *Howardella* | 0.63 | 0.52 | 0.66 | 0.57 | 0.08 | 0.78 | 0.38 | 0.58 |
| *Duncaniella* | 0.44 | 0.49 | 0.73 | 0.66 | 0.15 | 0.44 | 0.24 | 0.46 |
| *Massiliprevotella* | 0.57 | 0.62 | 0.43 | 0.55 | 0.11 | 0.81 | 0.55 | 0.93 |
| *Lentimicrobium* | 0.57 | 0.49 | 0.59 | 0.46 | 0.15 | 0.30 | 0.38 | 0.35 |
| *Herbinix* | 0.63 | 0.56 | 0.31 | 0.56 | 0.28 | 0.50 | 0.63 | 0.62 |
| *Faecalimonas* | 0.51 | 0.41 | 0.54 | 0.53 | 0.12 | 0.98 | 0.51 | 0.87 |
| *Flintibacter* | 0.40 | 0.79 | 0.27 | 0.52 | 0.23 | 0.45 | 0.12 | 0.44 |
| *Anaerovibrio* | 0.47 | 0.46 | 0.55 | 0.40 | 0.09 | 0.82 | 0.68 | 0.47 |
| *Rarimicrobium* | 0.38 | 0.57 | 0.4 | 0.33 | 0.11 | 0.72 | 0.21 | 0.22 |
| *Mogibacterium* | 0.38 | 0.32 | 0.54 | 0.34 | 0.07 | 0.99 | 0.32 | 0.99 |
| *Intestinibaculum* | 0.30 | 0.24 | 0.49 | 0.35 | 0.13 | 0.55 | 0.14 | 0.46 |
| *Saccharofermentans* | 0.48 | 0.27 | 0.29 | 0.3 | 0.08 | 0.19 | 0.46 | 0.63 |
| *Metaprevotella* | 0.32 | 0.32 | 0.37 | 0.33 | 0.09 | 0.49 | 0.37 | 0.50 |
| *Paraprevotella* | 0.37 | 0.34 | 0.27 | 0.33 | 0.09 | 0.29 | 0.41 | 0.48 |
| *Stomatobaculum* | 0.28 | 0.28 | 0.32 | 0.32 | 0.07 | 0.61 | 0.33 | 0.53 |
| *Syntrophococcus* | 0.27 | 0.27 | 0.21 | 0.29 | 0.10 | 0.96 | 0.37 | 0.91 |
| *Acetobacter* | 0.38 | 0.19 | 0.36 | 0.09 | 0.09 | 0.80 | 0.39 | 0.77 |
| *Marvinbryantia* | 0.24 | 0.14 | 0.28 | 0.21 | 0.05 | 0.61 | 0.19 | 0.44 |
| *Lactobacillus* | 0.40 | 0.15 | 0.12 | 0.18 | 0.10 | 0.07 | 0.43 | 0.46 |
| *Coprococcus* | 0.22 | 0.09 | 0.27 | 0.25 | 0.03 | 0.79 | < 0.01 | 0.06 |
| *Marseilla* | 0.21 | 0.22 | 0.25 | 0.11 | 0.06 | 0.47 | 0.60 | 0.79 |
| *Limosilactobacillus* | 0.26 | 0.11 | 0.17 | 0.13 | 0.07 | 0.30 | 0.14 | 0.45 |
| *Moryella* | 0.16 | 0.19 | 0.21 | 0.11 | 0.05 | 0.86 | 0.85 | 0.51 |
| *Fibrobacter* | 0.20 | 0.21 | 0.03 | 0.18 | 0.10 | 0.69 | 0.15 | 0.48 |
| *Alterileibacterium* | 0.19 | 0.07 | 0.17 | 0.15 | 0.04 | 0.22 | 0.12 | 0.36 |
| *Holdemania* | 0.16 | 0.04 | 0.18 | 0.04 | 0.09 | 0.65 | 0.96 | 0.57 |
| *Acinetobacter* | 0.15 | 0.12 | 0.06 | 0.07 | 0.05 | 0.24 | 0.45 | 0.61 |
| Solid Fraction |  |  |  |  |  |  |  |  |
| *Prevotella* | 28.7 | 31.8 | 32.1 | 30.2 | 2.33 | 0.26 | 0.61 | 0.44 |
| *Butyrivibrio* | 8.45 | 8.42 | 7.94 | 8.57 | 1.10 | 1.00 | 0.93 | 0.85 |
| *Treponema* | 8.27 | 5.25 | 8.69 | 5.96 | 0.74 | 0.31 | 0.49 | 0.92 |
| *Selenomonas* | 5.38 | 6.89 | 5.78 | 5.52 | 0.72 | 0.34 | 0.46 | 0.41 |
| *Megasphaera* | 5.56 | 6.23 | 4.80 | 6.18 | 1.57 | 0.99 | 0.72 | 0.88 |
| *Ruminococcus* | 5.58 | 4.66 | 6.08 | 6.32 | 0.27 | 0.86 | 0.03 | 0.06 |
| *Succiniclasticum* | 4.28 | 3.95 | 3.41 | 3.73 | 0.83 | 0.49 | 0.59 | 0.76 |
| *Schwartzia* | 2.52 | 2.55 | 2.33 | 2.82 | 0.27 | 0.85 | 0.92 | 0.55 |
| *Pseudobutyrivibrio* | 2.52 | 2.34 | 2.04 | 2.45 | 0.50 | 0.57 | 0.66 | 0.98 |
| *Acutalibacter* | 2.76 | 2.70 | 1.71 | 1.91 | 0.57 | 0.09 | 0.44 | 0.36 |
| *Acidaminococcus* | 1.97 | 2.00 | 1.84 | 2.11 | 0.43 | 1.00 | 0.76 | 0.90 |
| *Lachnobacterium* | 2.09 | 1.35 | 1.76 | 1.60 | 0.45 | 0.75 | 0.38 | 0.70 |
| *Olsenella* | 1.44 | 1.43 | 1.23 | 1.33 | 0.26 | 0.97 | 0.40 | 0.73 |
| *Dialister* | 1.23 | 1.29 | 1.56 | 1.31 | 0.47 | 0.70 | 0.24 | 0.07 |
| *Fibrobacter* | 1.55 | 0.84 | 1.30 | 0.87 | 0.43 | 0.80 | 0.59 | 0.85 |
| *Mailhella* | 1.12 | 1.07 | 0.95 | 1.25 | 0.36 | 0.88 | 0.93 | 0.66 |
| *Eubacterium* | 1.08 | 0.88 | 1.17 | 0.98 | 0.25 | 0.79 | 0.62 | 0.85 |
| *Mitsuokella* | 0.74 | 1.07 | 1.09 | 0.99 | 0.21 | 0.09 | 0.77 | 0.92 |
| *Saccharofermentans* | 1.15 | 0.95 | 0.76 | 0.98 | 0.17 | 0.27 | 0.68 | 0.48 |
| *Falsiporphyromonas* | 0.77 | 1.17 | 0.99 | 0.69 | 0.24 | 0.80 | 0.81 | 0.35 |
| *Sharpea* | 0.84 | 0.66 | 0.83 | 0.94 | 0.22 | 0.66 | 0.35 | 0.35 |
| *Anaerovibrio* | 0.75 | 0.94 | 0.64 | 0.78 | 0.15 | 0.82 | 0.30 | 0.55 |
| *Massiliprevotella* | 0.69 | 0.83 | 0.72 | 0.69 | 0.23 | 0.85 | 0.59 | 0.59 |
| *Bifidobacterium* | 0.53 | 0.56 | 0.31 | 1.32 | 0.38 | 0.81 | 0.73 | 0.93 |
| *Faecalimonas* | 0.56 | 0.56 | 0.86 | 0.75 | 0.34 | 0.90 | 0.98 | 0.71 |
| *Mediterranea* | 0.61 | 0.68 | 0.49 | 0.49 | 0.08 | 0.59 | 0.07 | 0.10 |
| *Succinivibrio* | 0.56 | 0.73 | 0.49 | 0.40 | 0.24 | 0.82 | 0.37 | 0.25 |
| *Clostridium_sensu_stricto* | 0.62 | 0.30 | 0.63 | 0.62 | 0.24 | 0.39 | 0.44 | 0.45 |
| *Mogibacterium* | 0.46 | 0.40 | 0.39 | 0.46 | 0.14 | 0.42 | 0.91 | 0.53 |
| *Stomatobaculum* | 0.52 | 0.39 | 0.31 | 0.42 | 0.09 | 0.18 | 0.38 | 0.93 |
| *Lentimicrobium* | 0.33 | 0.47 | 0.34 | 0.30 | 0.08 | 0.82 | 0.81 | 0.77 |
| *Pseudoramibacter* | 0.36 | 0.35 | 0.29 | 0.30 | 0.12 | 0.45 | 0.57 | 0.36 |
| *Metaprevotella* | 0.30 | 0.35 | 0.26 | 0.36 | 0.10 | 0.88 | 0.53 | 0.55 |
| *Herbinix* | 0.38 | 0.25 | 0.39 | 0.15 | 0.26 | 0.68 | 0.53 | 0.81 |
| *Syntrophococcus* | 0.25 | 0.42 | 0.19 | 0.29 | 0.08 | 0.54 | 0.02 | 0.27 |
| *Succinimonas* | 0.13 | 0.09 | 0.01 | 0.92 | 0.27 | 0.30 | 0.95 | 0.29 |
| *Pseudoscardovia* | 0.07 | 0.36 | 0.34 | 0.37 | 0.16 | 0.13 | 0.93 | 0.93 |
| *Catonella* | 0.24 | 0.13 | 0.39 | 0.35 | 0.08 | 0.98 | 0.02 | 0.09 |
| *Marvinbryantia* | 0.30 | 0.27 | 0.24 | 0.28 | 0.09 | 0.38 | 0.82 | 0.85 |
| *Ruminobacter* | 0.14 | 0.27 | 0.45 | 0.06 | 0.14 | 0.43 | 0.52 | 0.36 |
| *Paraprevotella* | 0.21 | 0.26 | 0.22 | 0.22 | 0.06 | 0.48 | 0.89 | 0.87 |
| *Intestinibaculum* | 0.19 | 0.14 | 0.23 | 0.33 | 0.12 | 0.90 | 0.58 | 0.59 |
| *Rarimicrobium* | 0.14 | 0.28 | 0.29 | 0.15 | 0.09 | 0.24 | 0.10 | 0.04 |
| *Lachnospira* | 0.18 | 0.17 | 0.34 | 0.16 | 0.06 | 0.97 | 0.57 | 0.71 |
| *Duncaniella* | 0.21 | 0.21 | 0.22 | 0.13 | 0.06 | 0.17 | 0.58 | 0.28 |
| *Coprococcus* | 0.22 | 0.17 | 0.19 | 0.17 | 0.05 | 0.71 | 0.75 | 0.94 |
| *Colidextribacter* | 0.21 | 0.14 | 0.18 | 0.22 | 0.06 | 0.84 | 0.63 | 0.42 |
| *Marseilla* | 0.18 | 0.22 | 0.17 | 0.16 | 0.07 | 0.84 | 0.85 | 0.68 |
| *Alterileibacterium* | 0.22 | 0.21 | 0.15 | 0.14 | 0.04 | 0.28 | 0.09 | 0.05 |
| *Clostridium_XlVa* | 0.13 | 0.17 | 0.16 | 0.18 | 0.03 | 0.27 | 0.91 | 0.94 |
| *Falcatimonas* | 0.16 | 0.15 | 0.15 | 0.16 | 0.03 | 0.84 | 0.28 | 0.30 |
| *Moryella* | 0.13 | 0.17 | 0.13 | 0.17 | 0.05 | 0.62 | 0.42 | 0.41 |
| *Aminicella* | 0.16 | 0.12 | 0.14 | 0.18 | 0.05 | 0.43 | 0.27 | 0.21 |
| *Adlercreutzia* | 0.13 | 0.11 | 0.17 | 0.15 | 0.04 | 0.67 | 0.92 | 0.92 |

^1^Experimental treatments: CON, control (experimental diet); MON, monensin (experimental diet plus 2.5μ*M* monensin sodium salt); CNSE100 (experimental diet plus 100 ppm CNSE granule); CNSE200 (experimental diet plus 200 ppm CNSE granule).

^2^Contrasts: ADD = CON vs. MON, CNSE100, CNSE200; MCN = MON vs. CNSE100, CNSE200; DOSE = CNSE100 vs. CNSE200.
